# A Novel Family of Cyst Proteins with Epidermal Growth Factor Repeats in *Giardia lamblia*

**DOI:** 10.1371/journal.pntd.0000677

**Published:** 2010-05-11

**Authors:** Pei-Wei Chiu, Yu-Chang Huang, Yu-Jiao Pan, Chih-Hung Wang, Chin-Hung Sun

**Affiliations:** Department of Parasitology, College of Medicine, National Taiwan University, Taipei, Taiwan, Republic of China; New York University School of Medicine, United States of America

## Abstract

**Background:**

*Giardia lamblia* parasitizes the human small intestine to cause diarrhea and malabsorption. It undergoes differentiation from a pathogenic trophozoite form into a resistant walled cyst form. Few cyst proteins have been identified to date, including three cyst wall proteins (CWPs) and one High Cysteine Non-variant Cyst protein (HCNCp). They are highly expressed during encystation and are mainly targeted to the cyst wall.

**Methodology and Principal Findings:**

To identify new cyst wall proteins, we searched the *G. lamblia* genome data base with the sequence of the *Cryptosporidium parvum* oocyst wall protein as a query and found an Epidermal Growth Factor (EGF)-like Cyst Protein (EGFCP1). Sequence analysis revealed that the EGF-like repeats of the EGFCP1 are similar to those of the tenascin family of extracellular matrix glycoproteins. EGFCP1 and HCNCp have a higher percentage of cysteine than CWPs, but EGFCP1 has no C-terminal transmembrane region found in HCNCp. Like CWPs and HCNCp, the EGFCP1 protein (but not transcript) was expressed at higher levels during encystation and it was localized to encystation-specific vesicles in encysting trophozoites. Like HCNCp, EGFCP1 was localized to the encystation-specific vesicles, cyst wall and cell body of cysts, suggesting that they may share a common trafficking pathway. Interestingly, overexpression of EGFCP1 induced cyst formation and deletion of the signal peptide from EGFCP1 reduced its protein levels and cyst formation, suggesting that EGFCP1 may help mediate cyst wall synthesis. We also found that five other putative EGFCPs have similar expression profiles and similar locations and that the cyst formation was induced upon their overexpression.

**Conclusions and Significance:**

Our results suggest that EGFCPs may function like cyst wall proteins, involved in differentiation of *G. lamblia* trophozoites into cysts. The results lead to greater understanding of parasite cyst walls and provide valuable information that helps develop ways to interrupt the *G. lamblia* life cycle.

## Author Summary

The biological goal of *Giardia lamblia* life cycle is differentiation into a cyst form (encystation) that can survive in the environment and infect a new host. Since cystic stages are key to transmission of parasites, this differentiation may be a target for interruption of the life cycle. Synthesis and assembly of the extracellular cyst wall are the major hallmarks of this important differentiation. During encystation, cyst wall structural proteins are coordinately synthesized and are mainly targeted to the cyst wall. However, only a few such proteins have been identified to date. In this study, we used a combination of bioinformatics and molecular approaches to identify new cyst structural proteins from *G. lamblia* and found a group of Epidermal Growth Factor (EGF)-like Repeats containing Cyst Proteins (EGFCPs). Interestingly, the levels of EGFCPs proteins increased significantly during encystation, which matches the characteristics of the *Giardia* cyst wall protein. Further characterization and localization studies suggest that EGFCPs may function like cyst wall proteins, involved in differentiation of *G. lamblia* trophozoites into cysts. Our results provide valuable information regarding the function of a new group of cyst proteins in parasite differentiation into cysts and help develop ways to interrupt the parasite life cycle.

## Introduction

*Giardia lamblia* is a prevalent intestinal parasite causing waterborne diarrheal disease [1], [2]. *G. lamblia* trophozoite causes malabsorption and diarrhea without penetrating intestinal epithelial cells [3]. It has two synchronous nuclei, moves by the flagella and adheres via the ventral disk to the upper intestine of host, a place suitable for their proliferation [2]. When a trophozoite is carried downstream to the lower intestine, encystation occurs, cyst wall is formed and both nuclei divide simultaneously, resulting in a cyst with four nuclei [2]. Transmission of giardiasis arises when cysts are ingested from faecally contaminated food or water.

The cyst form is the infective form capable of survival under hostile environments after excretion [2]. It has a resistant wall composed of proteins and polysaccharides to protect the parasite from hypotonic lysis by fresh water and from gastric acid during infection of the new host [4], [5]. The polysaccharide moiety is composed mainly of N-acetylgalactosamine homopolymer [6]. The strong interchain interactions of the polysaccharides and the strong interaction of the polysaccharide and the proteins may lead to a highly insoluble cyst wall [6].

The three known cyst wall proteins (CWPs) have similar expression levels, architectural motifs, and biological properties. Expression of the *cwp1-3* genes and a gene encoding an enzyme in the cyst wall polysaccharide biosynthetic pathway (glucosamine-6-phosphate isomerase-B, G6PI-B) increases with similar kinetics during encystation [7]–[11], suggesting the importance of gene regulation at transcriptional and/or translational level. During encystation, CWPs are concentrated within large membrane-bounded encystation secretory vesicles (ESVs) before transport to the cyst wall [7]–[9]. The ESVs have been proposed to be the *trans*-Golgi network or Golgi equivalents of the other eukaryotic cells [12], [13]. All three CWPs have N-terminal signal peptides, four to five tandem leucine-rich repeats (LRRs) and >14 positionally conserved cysteines [7]–[9]. Deletion of signal peptide or any one of LRRs prevented CWP3 from targeting to the ESVs or cyst wall [9]. Formation of intramolecular or intermolecular disulfide bonds between the cysteines may lead to heterooligomer formation between CWPs in the ESVs and cyst wall [9], [14]. Treatment of live encysting cells with DTT prevents the formation of disulfide bonds, ESVs and cyst wall [15], [13].

*G. lamblia* trophozoites are covered by variant surface proteins (VSPs) that are cysteine-rich type I integral membrane proteins, protecting them from protease and enzyme digestion in intestine [2], [16]. VSPs and CWPs have different expression patterns and subcellular localization profiles [17]. VSPs switch during vegetative growth or encystation and they are surface proteins that are transported to the trophozoite plasmalemma from endoplasmic reticulum (ER) pathway [17]. A High Cysteine Non-variant Cyst protein (HCNCp) has been identified recently [16]. It was originally annotated as a large VSP [16]. Like VSPs, it is a cysteine rich, acidic, type 1 integral membrane protein [16]. HCNCp has many CxC motifs that are rarely found in VSPs. Unlike CWPs, HCNCp has a higher molecular weight and many CxC or Cx2C motifs that are rarely found in CWPs and it does not have LRR motifs [7]–[9], [16]. Like CWPs, HCNCp is highly expressed during encystation and localized in ESVs of encysting trophozoites. However, HCNCp was localized to the wall and cell body of cysts, different from the exclusive cyst wall localization of the CWPs [16]. A total of 61 (0.63% of the genome) large High Cysteine Membrane (with a transmembrane region) proteins (HCMp) containing ≥400 amino acids, ≥10% Cys, ≥20 CxC and/or CxxC, but lacking the VSP-specific C-terminal CRGKA have been found in the *G. lamblia* genome [16]. Some of HCMps are recognized as VSP-like, EGF-like and transmembrane kinases-like [16].

A group of oocyst membrane proteins have been identified recently, including nine *Cryptosporidium* oocyst wall proteins (COWP1–9) in *Cryptosporidium parvum* and one COWP homolog (*Tg*COWP1) in *Toxoplasma gondii* [18], [19]. COWPs have signal peptides and two types of repeated domains but lack transmembrane regions [18], [19]. The type I and II domains both have 6 positionally conserved cysteines but the type I domain (65 amino acids) is longer than the type II domain (53 amino acids). All COWPs have the type I domains, but the type II domains are only present in COWP1–3 [18]. COWP1 is localized in the wall-forming bodies of late macrogametes and the inner oocyst wall of the double-walled oocysts, respectively [17]. COWP8 is localized in the oocyst wall [18]. The function of the cysteine-rich type I and II domains is not known, but it has been proposed that disulfide bonds of cysteine-rich protein may provide rigidity to the oocyst wall [17], [18]. COWP1–9 are up-regulated during later stages of *C. parvum* development [18].

To identify new cyst protein, we used the type I domain sequences of COWP8 to screen the G. lamblia genome data base (http://www.giardiadb.org/giardiadb/) [20]. We found a group of Epidermal Growth Factor (EGF)-like Cyst Proteins (EGFCPs) in the *G. lamblia* genome. Sequence analysis revealed that the EGF-like repeats of the EGFCPs are similar to those of the tenascin family glycoproteins. Tenascins are extracellular matrix glycoproteins that are highly expressed during embryonic cell development and reappear during wound repair, nervous regeneration and tumorigenesis [21]. Tenascins have multiple domains including the EGF-like repeats. A single EGF-like repeat contains ∼40 amino acids with six positionally conserved cysteines [22]. Nine EGF or EGF-like repeats are present in the EGFCP1. EGFCP1 matches some of the characteristics of HCNCp or HCMps, but it does not have a transmembrane region. Unlike CWPs and HCNCp, whose mRNA levels increased during encystation, the endogenous mRNA levels of the EGFCP1 decreased during encystation. Interestingly, the EGFCP1 protein levels significantly increased during encystation, which matches the characteristics of CWPs and HCNCp. Like CWPs, EGFCP1 formed polydisperse disulfide-bonded complexes detected in non-reducing gels and it was localized to ESVs in encysting trophozoites. Like HCNCp, EGFCP1 was localized to the wall and cell body of cysts (relatively little of EGFCP1 remained in the cyst cell body), suggesting that EGFCP1 and HCNCp may be involved in a novel secretory pathway for parasite cyst and for extracellular matrix assembly. We also found that overexpressed EGFCP1 induced the cyst formation. In addition, deletion of the signal peptide from EGFCP1 prevented its targeting to the ESVs and reduced its protein levels and cyst formation, suggesting that EGFCP1 may help mediate the cyst wall synthesis. By searching the *G. lamblia* genome data base (http://www.giardiadb.org/giardiadb/) [20], we found five other open reading frames with high similarity to EGFCP1. These five other putative EGFCPs were expressed in similar profiles and localized to similar locations and their overexpression resulted in an induction of cyst formation. Our results provide insights into the role of a novel group of cyst proteins with EGF repeats in parasite differentiation into cysts.

## Methods

### *G. lamblia* culture

Trophozoites of *G. lamblia* WB (ATCC 30957), clone C6, were cultured in modified TYI-S33 medium [23] and encysted as previously described [9]. Cyst count was performed on 24 h encysting cultures as previously described [24].

### Isolation and analysis of the *egfcp1* gene

The *G. lamblia* genome data base (http://www.giardiadb.org/giardiadb/) [20] was searched with the amino acid sequences of the type I domain of COWP8 (GenBank accession number **AY465056**) [19] using the BLASTP program [25]. Amino acid sequences with the highest similarity were found in an open reading frame with EGF-like repeats we named EGF-like Cyst Protein 1 (EGFCP1)(open reading frame 95162, GenBank accession number **XM_001704009**). The EGFCP1 coding region with 360 nt of 5′- flanking regions was cloned and the nucleotide sequence was determined. The *egfcp1* gene sequence in the data base was correct. To isolate the cDNA of the *egfcp1* gene, we performed RT-PCR with *egfcp1*-specific primers using total RNA from *G. lamblia*. For RT-PCR, 5 µg of DNase-treated total RNA from vegetative and 24 h encysting cells was mixed with oligo (dT)12–18 and Superscript II RNase H- reverse transcriptase (Invitrogen). Synthesized cDNA was used as a template in subsequent PCR with primers EGFCP1F (CACCATGATAGCCGCGGCCTTT) and EGFCP1R (CACACATCTACCATCGCG). Genomic and RT-PCR products were cloned into pGEM-T easy vector (Promega) and sequenced (Applied Biosystems, ABI).

### RNA extraction, RT-PCR and quantitative real time PCR analysis

Total RNA was extracted from *G. lamblia* cell line at the indicated differentiation stages using TRIzol reagent (Invitrogen). For RT-PCR, 5 µg of DNase-treated total RNA from vegetative and 24 h encysting cells was mixed with oligo (dT)12–18 and Superscript II RNase H- reverse transcriptase (Life Technologies). Synthesized cDNA (∼20 ng) was used as a template in subsequent PCR with 20–45 cycles (depending on the genes and primers). Semi-quantitative RT-PCR analysis of total *egfcp1* (open reading frame 95162, GenBank accession number **XM_001704009**), *egfcp1-ha*, endogenous *egfcp1*, *cwp1* (**U09330**), *ran* (**U02589**), glycyl t-RNA synthetase (open reading frame 9011, GenBank accession number XM_773334), *egfcp2* (open reading frame 113038, GenBank accession number XM_001707464, *egfcp3* (open reading frame 114815, GenBank accession number XM_001705096), *egfcp4* (open reading frame 16477, GenBank accession number XM_001710038), *egfcp5* (open reading frame 16322, GenBank accession number XM_001706828), and *egfcp6* (open reading frame 8687, GenBank accession number XM_001704393) gene expression was performed using primers egfcp1F and egfcp1R; egfcp13F and egfcp1HAR; egfcp1HAF and egfcp13R; cwp1F and cwp1R; ranF and ranR; glycylrealF and glycylrealR; egfcp2realF and egfcp2realR; egfcp3realF and egfcp3R; egfcp4realF and egfcp4R; egfcp5realF and egfcp5R; and egfcp6realF and egfcp6R, respectively. For quantitative real time PCR, SYBR Green PCR master mixture was used (Applied Biosystems). PCR was performed using an Applied Biosystems PRISMTM 7900 Sequence Detection System (Applied Biosystems). Specific primers were designed for detection of the total *egfcp1*, *egfcp1-ha*, endogenous *egfcp1*, *cwp1*, *ran*, glycyl t-RNA synthetase, *egfcp2*, *egfcp3*, *egfcp4*, *egfcp5*, and *egfcp6* genes: egfcp1realF and egfcp1realR; egfcp1HAF and egfcp1HAR; egfcp1HAF and egfcp13R; cwp1realF and cwp1realR; ranrealF and ranrealR; glycylrealF and glycylrealR; egfcp2realF and egfcp2realR; egfcp3realF and egfcp3realR; egfcp4realF and egfcp4realR; egfcp5realF and egfcp5realR; egfcp6realF and egfcp6realR. Two independently generated stably transfected lines were made from each construct and each of these cell lines was assayed three separate times. The results are expressed as relative expression level over control. Student's *t*-tests were used to determine statistical significance of differences between samples.

### Plasmid construction

All constructs were verified by DNA sequencing with BigDye Terminator 3.1 DNA Sequencing kit and an Applied Biosystems 3100 DNA Analyser (Applied Biosystems). Plasmid 5′Δ5N-Pac was a gift from Dr. Steven Singer and Dr. Theodore Nash [26]. Detailed cloning procedures for plasmids pPTEGFCP1, pNEGFCP1, pPTEGFCP1▵sp, and pPEGFCP2–6 are available as the supplementary materials (Fig. S1 and Table S1). Cloning steps include the use of plasmids pPop2NHA, pNLop2-1, and pPop2N [27], [28]. Plasmid ran32 has been previously described [28].

### Expression and purification of recombinant EGFCP1 protein

The genomic *egfcp1* gene was amplified using oligonucleotides egfcp1F and egfcp1R. The product was cloned into the expression vector pET Directional TOPO (Invitrogen) in frame with the C-terminal His and V5 tag to generate plasmid pEGFCP1. The pEGFCP1 plasmid was freshly transformed into *Escherichia coli* BL21 (DE3) pLysE (QIAexpressionist, Qiagen). An overnight pre-culture was used to start a 250-ml culture. *E. coli* cells were growth to an A600 of 0.5, and then induced with 1 mM isopropyl-D-thiogalactopyranoside (Promega) for 4 h. Bacteria were harvested by centrifugation and sonicated in 10 ml of buffer A (50 mM sodium phosphate, pH 8.0, 300 mM NaCl) containing 10 mM imidazole and protease inhibitor mixture (Sigma). The samples were centrifuged, and the supernatant was mixed with 1 ml of 50% slurry of nickel-nitrilotriacetic acid superflow (Qiagen). The resin was washed with buffer A containing 20 mM imidazole and eluted with buffer A containing 250 mM imidazole. Fractions containing EGFCP1 were pooled, dialyzed in 25 mM HEPES pH 7.9, 20 mM KCl, and 15% glycerol, and stored at −70°C. Protein purity and concentration were estimated by Coomassie Blue and silver staining compared with serum albumin. EGFCP1 was purified to apparent homogeneity (>95%).

### Generation of anti-EGFCP1 antibody

Recombinant EGFCP1 protein was purified from *E. coli* and used to generate rabbit polyclonal antibodies through a commercial vendor (Angene, Taipei, Taiwan).

### Transfection and Western blot analysis

Cells transfected with pN series plasmid were selected with G418 as described previously [29]. Stable transfectants were maintained at 150 µg/ml G418. Cells transfected with pP series plasmid containing the *pac* gene were selected and maintained with 54 µg/ml puromycin. Western blots were probed with anti-AU1 (1/5000 in blocking buffer; Covance) or anti-HA monoclonal antibody (1/5000 in blocking buffer; Sigma), and detected with horseradish peroxidase-conjugated goat anti-rabbit/mouse IgG (1/5000; Pierce) and enhanced chemiluminescence (GE Healthcare). Western blots were also probed with anti-EGFCP1 antibody (1/10000), anti-CWP1 antibody (1/10000) [30] or anti-human RAN antibody (1/5000) (Santa Cruz Biotechnology), and detected with horseradish peroxidase-conjugated goat anti-rabbit IgG (1/5000; Pierce) and enhanced chemiluminescence (GE Healthcare).

### Detecting EGFCPs in medium

5′Δ5N-Pac, pPTEGFCP1▵sp, and pPTEGFCP1–6 cell lines were cultured in growth medium or encystation medium for 24 h. The cultured media were collected and centrifuged at 800×g for 10 min and then passed through a 0.22 µm syringe filter to eliminate *G. lamblia* cells. Four ml of the collected medium was incubated with 40 µl of anti-HA antibody conjugated to beads (Bethyl Laboratories Inc.). The beads were washed three times with 500 µl of luciferase lysis buffer (Promega). Finally the beads were then resuspended in sample buffer and analyzed by Western blot and probed with anti-HA monoclonal antibody (1/5000 in blocking buffer; Sigma).

### Immunofluorescence assays

Stably transfected cells were harvested after 24 h in growth or encystation medium under drug selection, washed in phosphate-buffered saline, and attached to glass coverslips (2×10^6^ cells/coverslip) and then fixed and stained [11]. The cysts from encystation medium were treated with water for 5 times. Cells were reacted with anti-CWP1 [30] or anti-HA monoclonal antibody (1/300 in blocking buffer; Sigma), and anti-rabbit ALEXA 568 or anti-mouse ALEXA 488 (1/500 in blocking buffer; Molecular Probes) as the detector. The ProLong antifade kit with 4′,6-diamidino-2-phenylindole (DAPI) (Invitrogen) was used for mounting. Images were acquired using a Leica TCS SP2 Spectral Confocal System.

## Results

### Identification and characterization of the *egfcp1* gene

**Figure 1:**
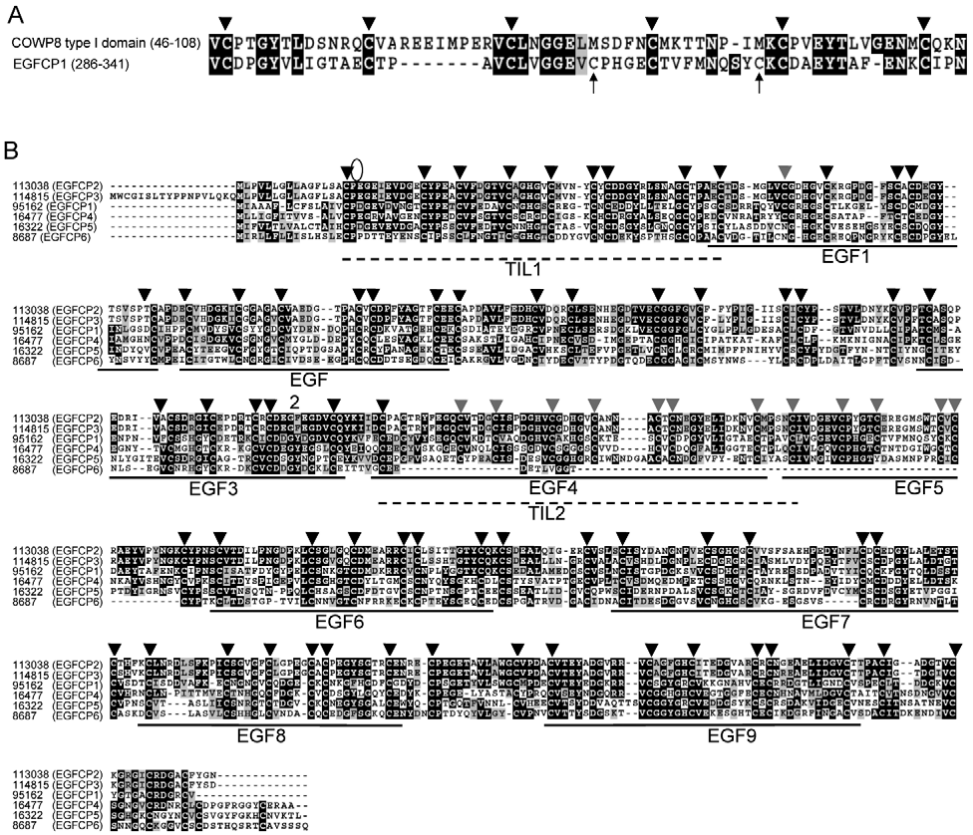
Sequence analysis of EGFCPs. (A) Alignment of the amino acid sequences of the type I domain of COWP8 and part of giardial EGFCP1. To identify possible new cyst wall proteins, we queried the *G. lamblia* genome data base (http://www.giardiadb.org/giardiadb/) [20] with the amino acid sequences of the type I domain of COWP8 (residues 46–108, GenBank accession number AY465056) [19] using the BLASTP algorithm [25]. This search identified similarity of the type I domain of COWP8 and part of giardial EGFCP1 (residues 286–341 of open reading frame number 95162 in the *G. lamblia* genome data base) (∼36% identity and ∼47% similarity). Numbers indicate positions of the residues relative to the first amino acid. Amino acids that are similar or identical to the consensus according to Clustal W 1.83 [49] are indicated in gray or black. Six positionally conserved cysteines are shown with black triangles. Additional cysteines in EGFCP1 are pointed by arrows. (B) Sequence alignment of EGFCPs. We searched the *G. lamblia* genome data base (http://www.giardiadb.org/giardiadb/) [20] using the amino acid sequences of EGFCP1 (open reading frame number 95162 in the *G. lamblia* genome data base). Amino acid sequences with similarity to that of EGFCP1 were found in five other open reading frames including 113038, 114815, 16477, 16322 and 8687. Amino acids that are similar or identical to the consensus Clustal W 1.83 [49] are indicated in gray or black. The predicted signal peptide cleavage site of the EGFCP1 (Signal P prediction) [34] is between residues 16 and 17 as indicated by an open oval. The location of nine EGF-like repeats of EGFCP1 predicted by SMART analysis (http://smart.embl-heidelberg.de) is underlined. The location of two TIL domains of EGFCP1 predicted by pfam analysis (http://pfam.sanger.ac.uk/) is indicated by dotted lines. Seventy to eighty-three cysteines are present in these EGFCPs (also see Fig. 2). Sixty-seven cysteines of all EGFCPs are positionally conserved (black triangles). Additional thirteen cysteines are positionally conserved in at least four EGFCPs (gray triangles).

To identify possible new cyst wall proteins, we queried the *G. lamblia* genome data base (http://www.giardiadb.org/giardiadb/) [20] with the amino acid sequences of the type I domain of COWP8 (GenBank accession number **AY465056**) [19]. COWPs have been localized in the wall-forming bodies of late macrogametes and the inner oocyst wall of the double-walled oocysts, respectively [18], [19]. Amino acid sequences with similarity to the type I domain of COWP8 were found in an open reading frame with EGF-like repeats and we named it EGF-like Cyst Protein 1 (EGFCP1) (open reading frame 95162, GenBank accession number **XM_001704009**) (Figs. 1A and 1B). A typical type I domain of COWP8 has 6 cysteines spaced 10 to 12 amino acids apart (Fig. 1A) [18], [19]. The 6 cysteines in the type I domain of COWP8 are positionally conserved with some cysteine residues in EGFCP1, but EGFCP1 has two additional cysteine residues (Fig. 1A).

AT-rich initiator sequences that have been found spanning the transcription start sites of many genes [31], [32] are also present in the 5′- flanking region of the *egfcp1* gene (−27 to −1 relative to translation start site) (data not shown). Classic Myb2 binding sites “C(T/A)ACAG” have not been found in the 200-bp 5′-flanking region of the *egfcp1* gene [28]. A classic polyadenylation signal (ATGTAAAC) [33] was 20 nt downstream of the stop codon (data not shown). Comparison of genomic and cDNA sequences showed that the *egfcp1* gene contained no introns. Short untranslated regions and lack of introns are typical of giardial transcripts [20]. The deduced EGFCP1 protein contains 572 amino acids with a predicted molecular mass of ∼61.8 kDa and an acidic pI 4.12 (Figs. 1B and S1A). The N-terminal 16 amino acids of EGFCP1 comprise a predicted signal peptide sequence (Signal P prediction) [34] with the most likely cleavage site between positions 16 and 17 (Fig. 1B).

Because VSPs, HCNCp and EGFCP1 have common characteristics, including high cysteine contents, acidic pIs, and N-termianl signal peptides, they were compared in more details. A well-studied VSP, TSA417 [17], was used as a representative of VSPs in this work. The C-terminal sequence of VSPs, HCNCp and EGFCP1 are all divergent (Fig. S2A, “CRGKA”, “CCRRSKAV” and “CRDGRCV”). HCNCp and EGFCP1 have no GGCY and zinc finger motifs which are found in VSPs [16]. In addition, HCNCp and EGFCP1 have common characteristics that are not present in VSPs. HCNCp and EGFCP1 have 8–11 “CxC” and 33–36 “Cx4-6C” motifs, but TSA417 has only 1 “CxC” and 9 “Cx4-6” motifs. EGFCP1 does not have some characteristics of VSPs and HCNCp. For example, VSPs and HCNCp have C-terminal transmembrane regions, but EGFCP1 does not have a transmembrane domain as predicted by TMHMM (http://www.cbs.dtu.dk/services/TMHMM/) (data not shown and Fig. S2A). TSA417 (VSP) and HCNCp have 29 and 77 “Cx2C” motifs, respectively, but EGFCP1 has only 2 “Cx2C” motifs (Fig. S2B). TSA417 (VSP) and HCNCp have >5 “Cx3C” but EGFCP1 does not have this motif. EGFCP1 matches some of the characteristics of HCNCp or HCMps, which contain ≥400 amino acids and ≥10% Cys, but it only has 11 CxC and 2 CxxC and does not match the characteristics of ≥20 CxC and/or CxxC (Figs. S2A and S2B) [16].

A query against the genome of all organisms using the NCBI BLAST web server using the amino acid sequences of the EGFCP1 suggests that the EGF-like repeats of the EGFCP1 are similar to those of the tenascin family glycoproteins in mammals. Tenascins are extracellular matrix glycoproteins expressed in embryonic cell development, tissue repair, and tumor stroma [21], [35]. They can function in inhibiting cell adhesion, enhancing migration and invasion of cancer cells, and promoting cancer cell proliferation [21]. They have multiple domains including the EGF-like repeats. A single EGF-like repeat contains ∼40 amino acids with six positionally conserved cysteines [22]. These repeats have been found in extracellular proteins and in many extracellular domains of membrane-bound proteins that function as sensors or receptors or are involved in extracellular matrix remodeling [22], [36], [37]. EGFCP1 has nine EGF or EGF-like repeats predicted by SMART analysis (http://smart.embl-heidelberg.de) (Figs. 1B and S3). In addition, EGFCP1 also has two putative trypsin inhibitor-like (TIL) cysteine rich domains (Pfam: PF01826) as predicted by pfam (http://pfam.sanger.ac.uk/search) (Figs. 1B and S4). An EGF-like repeat is overlapped with the second TIL domain (Fig. 1B). TIL domains have been found in some extracellular proteins or small serine protease inhibitors that inhibit peptidases in parasitic nematodes, arthropods and amphibian [38]–[40]. The TIL domain has 10 positionally conserved cysteine residues forming 5 disulfide bonds [41]. The similarity between the TIL domains of EGFCP1 and those of two *Ancylostoma caninum* anti-coagulant precursors, one *A. ceylanicum Ascaris*-type serine protease inhibitor, and one *Apis mellifera* (honeybee) chymotrypsin inhibitor is mainly limited to the cysteines residues. Each of the two putative TIL domains of EGFCP1 has 9 cysteines while one more cysteine can be found outside of the TIL domain (see inserted sequence in Fig. S4).

**Figure 2:**
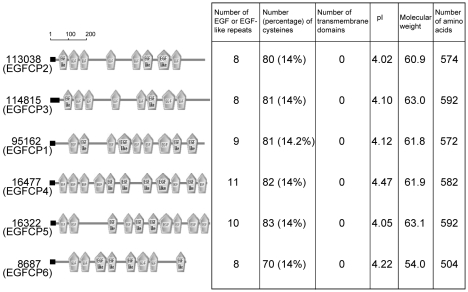
Summary of domain characteristics in EGFCPs. The number and location of the EGF or EGF-like repeats in EGFCPs are predicted by SMART analysis (http://smart.embl-heidelberg.de). Eight to eleven EGF or EGF-like repeats are present in these EGFCPs. Seventy to eighty-three cysteines are present in these EGFCPs. They have no transmembrane domains as predicted by TMHMM (http://www.cbs.dtu.dk/services/TMHMM/). They have acidic pIs and signal peptides (black boxes) as predicted by Signal P [34].

We further searched the *G. lamblia* genome data base (http://www.giardiadb.org/giardiadb/) [20] using the amino acid sequences of the EGFCP1. Amino acid sequences with similarity to that of EGFCP1 were found in five other open reading frames including 113038 (EGFCP2), 114815 (EGFCP3), 16477 (EGFCP4), 16322 (EGFCP5) and 8687 (EGFCP6) (Fig. 1B). Seventy to eighty-three cysteines are present in these EGFCPs (Fig. 2). Interestingly, sixty-seven cysteines of all EGFCPs are positionally conserved (Fig. 1B). Additional thirteen cysteines are positionally conserved in at least four EGFCPs (Fig. 1B). All EGFCPs have 8–11 EGF or EGF-like repeats (Fig. 2). All EGFCPs have acidic pIs and signal peptides (Signal P prediction) [34] but they have no transmembrane domains as predicted by TMHMM (http://www.cbs.dtu.dk/services/TMHMM/) (Fig. 2).

### Encystation-induced expression of EGFCP1

**Figure 3:**
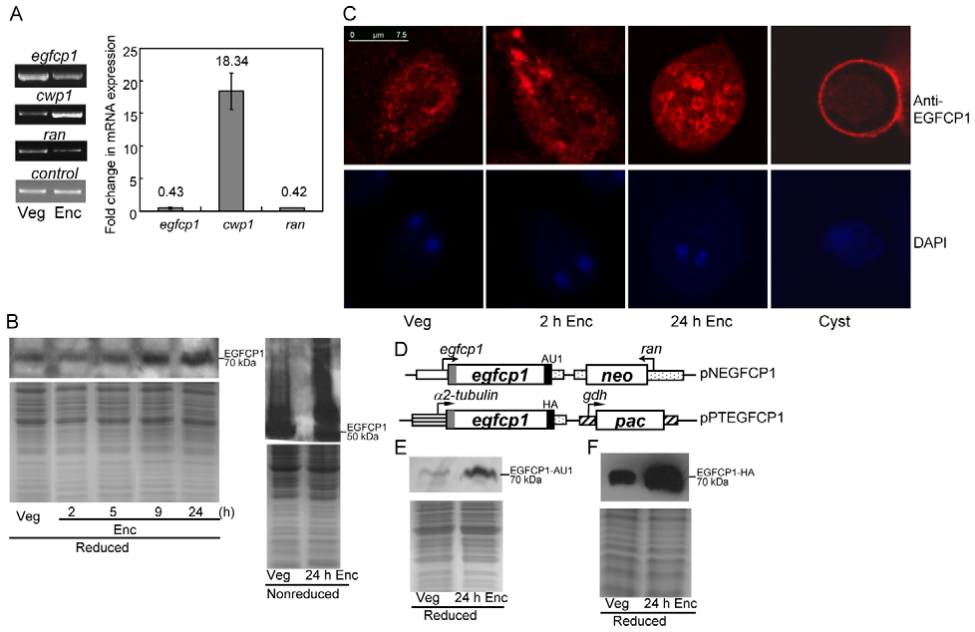
Analysis of *egfcp1* gene expression. (A) RT-PCR and quantitative real time PCR analysis of *egfcp1* gene expression. RNA samples were prepared from *G. lamblia* wild-type non-transfected WB cells cultured in growth (Veg) or encystation medium and harvested at 24 h (Enc). RT-PCR and real time PCR were preformed using primers specific for *egfcp1*, *cwp1*, *ran*, and glycyl t-RNA synthetase genes. Representative results are shown on the left panel. Transcript levels of specific genes were normalized to glycyl t-RNA synthetase (control) transcript levels [50]. Fold changes in mRNA expression are shown as the ratio of transcript levels in encysting cells relative to vegetative cells. Results are expressed as the means ± S.E. of at least three separate experiments (right panel). (B) EGFCP1 protein levels in different stages. The wild-type non-transfected WB cells were cultured in growth (Veg) or encystation medium (Enc) for 2, 5, 9, and 24 h and then subjected to SDS-PAGE and Western blot (left). The blot was probed by anti-EGFCP1 antibody. Representative results are shown. The results from Western blot analysis of nonreduced proteins are shown at right panel. Equal amounts of proteins loaded were confirmed by SDS-PAGE and Coomassie blue staining (lower panels). (C) Localization of EGFCP1. The wild-type non-transfected WB cells were cultured in growth (Veg) or encystation medium (Enc) and harvested at 2 h or 24 h, and then subjected to immunofluorescence analysis using anti-EGFCP1 antibody (1/300) for detection (upper panels). The lower panels show the DAPI staining of cell nuclei. EGFCP1 was localized to the ER in a vegetative trophozoite (Veg). During encystation, EGFCP1 was localized to the ER and some big vesicles in a 2 h encysting trophozoite (2 h Enc) and to the ESVs in a 24 h encysting trophozoite (24 h Enc). In the cyst stage, EGFCP1 was localized to the cyst wall and weakly to cell body (Cyst). Most (>80%) cells or cysts are positive stained. (D) The pNEGFCP1 and pPTEGFCP1 plasmids. A *neo* or *pac* gene is under the control of the 5′- and 3′-flanking regions of the *ran* (dotted box) or *gdh* (slashed box) gene. The *egfcp1* gene is under the control of its own 5′-flanking region (open boxes) or constitutively expressed *α2-tubulin* promoter (striated box) and the 3′-flanking region of the *ran* gene (dotted box). The filled black box indicates the coding sequence of the AU1 (for pNEGFCP1 plasmid) or HA (for pPTEGFCP1 plasmid) epitope tag. The filled gray box indicates the coding sequence of the signal peptide. The arrows show the directions of gene transcription. (E) EGFCP1 protein levels in pNEGFCP1 stable transfectants. The pNEGFCP1 stable transfectants were cultured in growth medium (Veg) or encystation medium and harvested at 24 h (Enc). AU1-tagged EGFCP1 protein was detected using an anti-AU1 antibody by Western blot analysis of reduced proteins. Coomassie-stained total protein loading control is shown below. (F) EGFCP1 protein levels in pPTEGFCP1 stable transfectants. The pPTEGFCP1 stable transfectants were cultured in growth medium (Veg) or encystation medium and harvested at 24 h (Enc). HA-tagged EGFCP1 protein was detected using an anti-HA antibody by Western blot analysis of reduced proteins. Coomassie-stained total protein loading control is shown below.

RT-PCR and quantitative real time PCR analysis of total RNA showed that the native *egfcp1* transcript was present in vegetative cells and decreased significantly during encystation (Fig. 3A). The *cwp1* and *ran* genes, which encode a component of the cyst wall [7] and a *ras*-related nuclear protein [29], were upregulated and downregulated during encystation, respectively, as previously reported [42].

To determine the expression of EGFCP1 protein, we purified the full-length recombinant EGFCP1 from *E. coli* and used it to generate an antibody specific to EGFCP1 (Fig. S5A for purified EGFCP1). Western blot analysis confirmed that this antibody recognized the EGFCP1 protein at a size of ∼70 kDa (Figs. 3B and S5B). EGFCP1 was expressed in vegetative cells and its levels increased significantly during encystation (Fig. 3B). Compared with the expression profile of EGFCP1, the levels of CWPs increase more during encystation [9]. When the samples were not reduced, EGFCP1 was expressed as a smear with a molecular mass ranging above ∼50 kDa band, indicating the formation of larger complexes through intermolecular disulfide linkages (Fig. 3B). CWPs formed similar polydisperse disulfide-bonded complexes [7]–[9]. Immunofluorescence assays showed that EGFCP1 was localized to the ER in vegetative trophozoites (Fig. 3C, Veg). During early encystation, EGFCP1 was localized to the ER and some big vesicles in encysting trophozoites (Fig. 3C, 2 h Enc). During late encystation, EGFCP1 was localized to the ESVs in encysting trophozoites (Fig. 3C, 24 h Enc). In the cyst stage, EGFCP1 was localized to the cyst wall and weakly to cell body (Fig. 3C, Cyst). It seems that relatively little of EGFCP1 remained in the cyst cell body as compared with the results of HCNCp [16].

To determine the expression of EGFCP1 protein, we prepared construct pNEGFCP1 in which the *egfcp1* gene is controlled by its own promoter and contains an AU1 epitope tag at its C terminus (Fig. 3D) and stably transfected it into *G. lamblia*. A ∼70-kDa protein was detected (Fig. 3E), which is slightly larger than the predicted ∼61.8-kDa molecular mass of EGFCP1 with the AU1 tag (∼0.8 kDa). The EGFCP1 protein levels increased significantly during encystation (Fig. 3E). However, the *egfcp1-au1* mRNA levels decreased significantly in encysting pNEGFCP1 transfectants, similar to the results in wild-type cells (data not shown, see Fig. 3A for wild-type cells). The lack of correlation between the steady state mRNA and protein levels could be due to an increase in translation rate or protein half life during encystation.

The AU1-tagged EGFCP1 in the pNEGFCP1 cell line can be detected by immunofluorescence assays but the signal was too weak to be observed (data not shown). We therefore prepared construct pPTEGFCP1 in which the *egfcp1* gene is controlled by an *α2-tubulin* promoter and has an HA epitope tag at its C terminus (Fig. 3D). The *α2-tubulin* gene is down-regulated during encystation [24]. We also found that the HA-tagged *egfcp1* mRNA levels decreased significantly in encysting pPTEGFCP1 transfectants (data not shown). However, the levels of the HA-tagged EGFCP1 protein also increased significantly during encystation (Fig. 3F).

### Localization of EGFCP1

**Figure 4:**
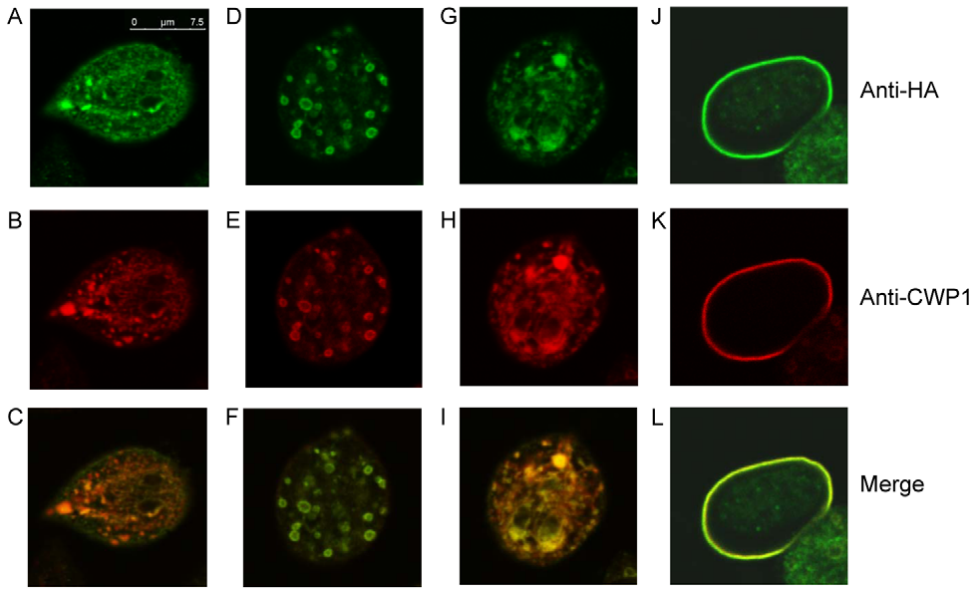
Localization of EGFCP1. The pPTEGFCP1 stable transfectants were cultured in growth medium (Veg) or encystation medium, harvested at 24 h (Enc), and then subjected to immunofluorescence analysis using anti-HA and anti-CWP1 antibody for detection of EGFCP1-HA and CWP1 proteins. Panels A and B show that EGFCP1-HA and CWP1 localizes to the ER in a vegetative trophozoite, respectively. Panels D and E show that EGFCP1-HA and CWP1 localizes to the ESVs in an encysting trophozoite, respectively. Panels G and H show that EGFCP1-HA and CWP1 localizes to the ER and some big vesicles in an encysting trophozoite, respectively. Panel J shows that EGFCP1-HA localizes to the cyst wall and weakly to the cell body in a cyst. Panel K shows that CWP1 localizes to the cyst wall of the same cyst. Panel C, F, I, or L is the merged images of A and B, D and E, G and H, or J and K, respectively.

Immunofluorescence assays showed that the HA-tagged EGFCP1 was localized to the ER in vegetative trophozoites (Fig. 4A). During encystation, EGFCP1-HA was localized to the ESVs in encysting trophozoites (Fig. 4D). It was also localized to the ER and some big vesicles in a few positive encysting cells (Fig. 4G). In these stages, CWP1 was also colocalized with EGFCP1 (Figs. 4A–I). In the cyst stage, CWP1 was localized to the cyst wall but EGFCP1 was localized to the cyst wall and weakly to the cell body (Figs. 4J–L). The number of EGFCP1-HA positively stained cells detected in vegetative trophozoites was small (20%) and increased (to ∼80%) during encystation.

**Figure 5:**
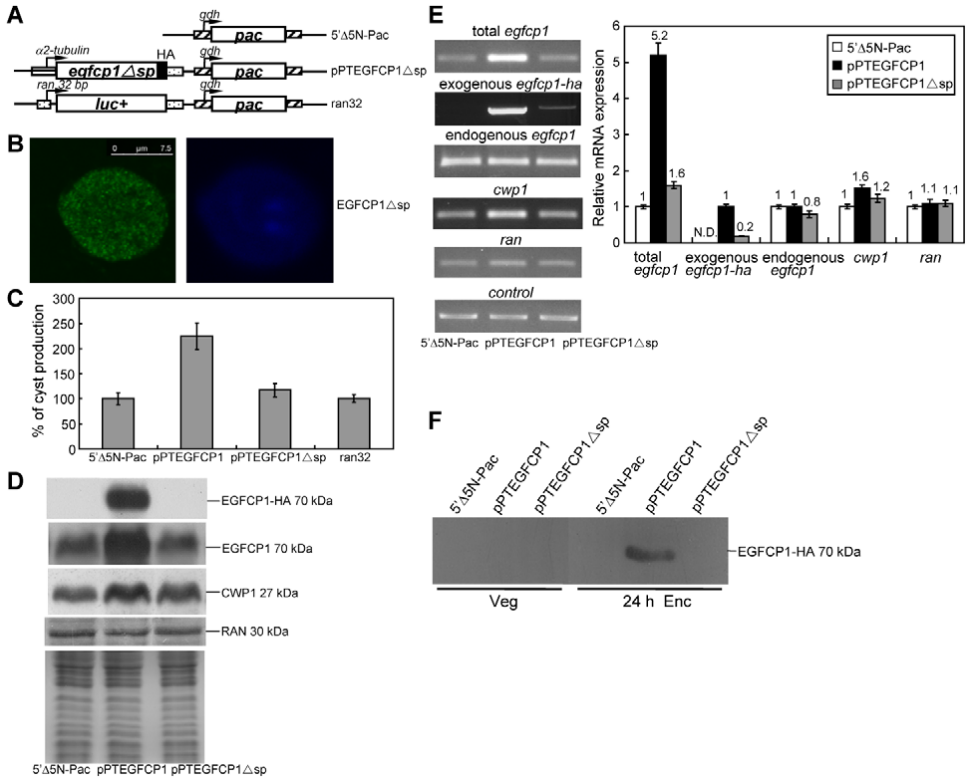
Overexpression of EGFCP1 increased the levels of cyst formation. (A) Diagrams of the 5′▵5N-Pac, ran32, and pPTEGFCP1▵sp plasmid. The expression cassettes of the *pac* and *egfcp1* genes (open box) are the same as in Fig. 3D. The ran32 plasmid constains a luciferase gene under the control of the 32 bp *ran* promoter (dotted box). The pPTEGFCP1▵sp plasmid contains an *egfcp1* gene lacking the coding sequence for the predicted signal peptide sequence (nucleotides 4–48) (gray box in Fig. 3D). (B) Localization of an EGFCP1 mutant with a deletion of signal peptide (EGFCP1▵sp). The pPTEGFCP1▵sp stable transfectants were cultured in encystation medium and harvested at 24 h, and then subjected to immunofluorescence analysis using anti-HA antibody for detection (left panel). The right panel shows the DAPI staining of cell nuclei. (C) Cyst count. The 5′▵5N-Pac, pPTEGFCP1 and pPTEGFCP1▵sp stable transfectants were cultured in encystation medium for 24 h, treated with water for 5 times, and subjected to cyst count. The sum of total cysts is expressed as relative expression level over control. Values are shown as means ± S.E. (D) Deletion of signal peptide reduced the levels of the EGFCP1 protein. The 5′▵5N-Pac, pPTEGFCP1, and pPTEGFCP1▵sp stable transfectants were cultured in encystation medium for 24 h and then subjected to SDS-PAGE and Western blots. The blots were probed by anti-HA, anti-EGFCP1, anti-CWP1, and anti-RAN antibodies. Equal amounts of proteins loaded were confirmed by detection of giardial RAN protein and Coomassie-stained total protein loading control (in the bottom panel). Representative results are shown. (E) Deletion of signal peptide reduced the levels of the *egfcp1* transcripts. The 5′▵5N-Pac, pPTEGFCP1, and pPTEGFCP1▵sp stable transfectants were cultured in encystation medium for 24 h and then subjected to RT-PCR (left panel) and quantitative real time PCR analysis (right panel). RT-PCR was preformed using primers specific for total *egfcp1*, exogenous *egfcp1-ha*, endogenous *egfcp1*, *cwp1*, *ran*, and glycyl t-RNA synthetase genes. Real time PCR was preformed using primers specific for total *egfcp1*, exogenous *egfcp1-ha*, endogenous *egfcp1*, *cwp1*, *ran* and glycyl t-RNA synthetase genes. Transcript levels of specific genes were normalized to glycyl t-RNA synthetase (control) transcript levels [50]. Fold changes in mRNA expression are shown as the ratio of transcript levels in pPTEGFCP1 or pPTEGFCP1▵sp cell line relative to the 5′▵5N-Pac cell lines. Results are expressed as the means ± S.E. of at least three separate experiments. N.D.  =  Not determined. (F) Release of EGFCP1 into medium during encystation. 5′Δ5N-Pac, pPTEGFCP1, and pPTEGFCP1▵sp stable transfectants were cultured in vegetative growth medium (Veg) or encystation medium (Enc) for 24 h. The cultured media were immunoprecipitated with the anti-HA antibody and then analyzed by Western blot using anti-HA antibody.

To further understand the function of EGFCP1, we constructed a pPTEGFCP1▵sp plasmid which encodes a mutant EGFCP1 (EGFCP1▵sp) lacking the predicted N terminal signal peptide (residues 2–16) (Fig. 5A). We found that deletion of the predicted N terminal signal peptide (residues 2–16, construct pPTEGFCP1▵sp, Fig. 5A) decreased the number of positively stained cells (from 80%) to 10% during encystation. The staining was weak and limited to cytosol and some small vesicles (Fig. 5B) and no cysts were observed to be positively stained (data not shown), suggesting that the N terminal signal peptide sequence is essential to target EGFCP1 to the ESVs, and possibly to the cyst wall.

### Overexpression of EGFCP1 increased the levels of cyst formation

We further investigated the effect of EGFCP1 on cyst formation using the EGFCP1 overexpressing cell line. The Protein levels of the endogenous EGFCP1 plus vector expressed EGFCP1-HA in the EGFCP1 overexpressing cell line increased significantly (Fig. 5D, EGFCP1) relative to the control cell line which expressed only the puromycin selection marker (5′Δ5N-Pac) (Fig. 5A) [26]. We found that the cyst number in the EGFCP1 overexpressing cell line (pPTEGFCP1, in which the *egfcp1* gene is controlled by an *α2-tubulin* promoter) increased by ∼2.3-fold (*p*<0.05) relative to the control cell line which expresses only the puromycin selection marker (5′▵5N-Pac) (Figs. 5A and C), indicating that the overexpressed EGFCP1 can increase the cyst formation. The cyst number in the luciferase overexpressing cell line did not change (ran32)(Figs. 5A and C). We also found that deletion of the predicted N terminal signal peptide of EGFCP1 (residues 2–16, construct pPTEGFCP1▵sp) decreased cyst number by ∼50% (*p*<0.05) relative to the levels in the pPTEGFCP1 cell line during encystation (Figs. 5A and C). We further analyzed whether the protein levels of the EGFCP1▵sp were changed. As shown by Western blot analysis, the levels of EGFCP1▵sp decreased significantly compared with that of wild type EGFCP1 during encystation (Fig. 5D). The HA-tagged EGFCP1▵sp protein can be detected with a longer exposure (Fig. S5C). These results indicate that EGFCP1 can increase the cyst formation and deletion of the signal peptide from EGFCP1 reduced its protein levels and cyst formation.

We further analyzed whether the transcript levels of the EGFCP1▵sp were changed. As shown by RT-PCR and quantitative real time PCR analysis, the levels of HA-tagged *egfcp1▵sp* mRNA decreased significantly compared with that of wild type HA-tagged *egfcp1* during encystation (Fig. 5E, exogenous *egfcp1-ha*). We did not detect any HA-tagged *egfcp1* transcripts in the 5′▵5N-Pac control cell line. In addition, the mRNA levels of the endogenous *egfcp1* plus vector expressed *egfcp1-ha* in the EGFCP1 or EGFCP1▵sp overexpressing cell line increased by ∼5 or ∼1.6-fold (*p*<0.05) (Fig. 5E, total *egfcp1*) relative to the control cell line which expressed only the puromycin selection marker (5′Δ5N-Pac) (Fig. 5A) [26]. We also found that the levels of the CWP1 protein and mRNA increased with the increase of the cyst counts in the EGFCP1 overexpressing cell line (Fig. 5D and E). As a control, the levels of the endogenous RAN protein and mRNA in the EGFCP1 or EGFCP1▵sp overexpressing cell line did not change relative to the 5′Δ5N-Pac control cell line (Fig. 5D and E).

### Release of EGFCP1 into medium during encystation

We further tried to understand whether EGFCP1 can be released into medium. The EGFCP1 overexpressing cell line (pPTEGFCP1) was cultured in growth or encystation medium for 24 h. Both culture media were then immunoprecipitated with anti-HA antibody and then subjected to Western blot analysis. Intact EGFCP1 (a ∼70-kDa band, which is slightly larger than the predicted ∼61.8-kDa molecular mass of EGFCP1 with ∼1 kDa HA tag) was detected in the encystation medium, whereas there was no evidence for EGFCP1 release into the normal growth medium (Fig. 5F). We did not detect any released EGFCP1▵sp in the culture medium of the EGFCP1▵sp cell line and vector control cell line (Fig. 5F). The results suggest that EGFCP1 can be released into medium during encystation.

### Encystation-induced expression of EGFCP2–6

**Figure 6:**
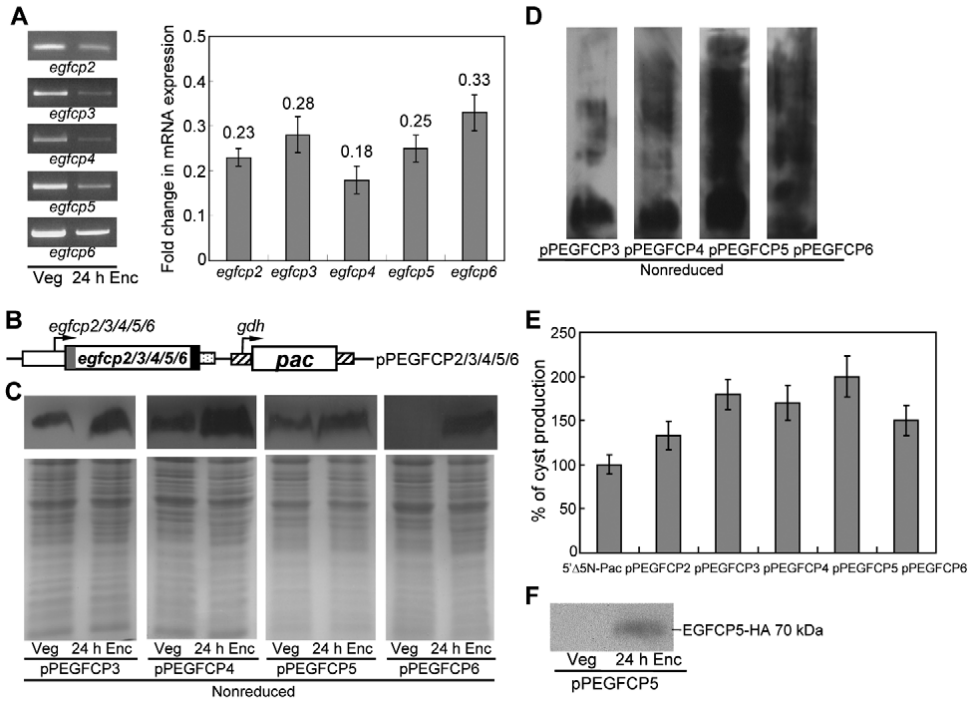
Overexpression of EGFCP2–6 increased the levels of cyst formation. (A) Analysis of *egfcp2–6* gene expression. RNA samples were prepared from *G. lamblia* wild-type non-transfected WB cells cultured in growth (Veg) or encystation medium and harvested at 24 h (Enc). RT-PCR and real time PCR were preformed using primers specific for *egfcp2–6* and glycyl t-RNA synthetase genes. Representative results are shown on the left panel. Transcript levels of specific genes were normalized to glycyl t-RNA synthetase (control) transcript levels [50]. Fold changes in mRNA expression are shown as the ratio of transcript levels in encysting cells relative to vegetative cells. Results are expressed as the means ± S.E. of at least three separate experiments (right panel). (B) Diagrams of the pPEGFCP2 (or pPEGFCP3, pPEGFCP4, pPEGFCP5, and pPEGFCP6) plasmid. The expression cassette of the *pac* gene (open box) is the same as in Fig. 3D. The *egfcp2* (or *egfcp3*, *egfcp4*, *egfcp5*, *egfcp6*) gene is under the control of its own 5′-flanking region (open boxes) and the 3′-flanking region of the *ran* gene (dotted box). The filled black box indicates the coding sequence of the HA epitope tag. (C) Protein levels of EGFCP2–6 in different stages. The pPEGFCP2–6 stable transfectants were cultured in growth medium (Veg) or encystation medium and harvested at 24 h (Enc). HA-tagged EGFCP2–6 proteins were detected using an anti-HA antibody by Western blot analysis of reduced proteins. Their size are very similar, ∼70 kDa, except that the HA-tagged EGFCP2 protein was not detected (data not shown). Coomassie-stained total protein loading control is shown below. Representative results are shown. (D) Western blot analysis of nonreduced EGFCP2–6 proteins. HA-tagged EGFCP2–6 proteins were detected using an anti-HA antibody by Western blot analysis of nonreduced proteins. Their pattern are similar, except that the HA-tagged EGFCP2 protein was not detected (data not shown). (E) Cyst count. The 5′▵5N-Pac, pPEGFCP2–6 stable transfectants were cultured in encystation medium for 24 h and then subjected to cyst count. The sum of total cysts is expressed as relative expression level over the control (5′▵5N-Pac). Values are shown as means ± S.E. (F) Secretion of EGFCP5 into medium during encystation. pPTEGFCP5 stable transfectants were cultured in vegetative growth medium (Veg) or encystation medium (Enc) for 24 h. The cultured media were immunoprecipitated with the anti-HA antibody and then analyzed by Western blot using anti-HA antibody.

**Figure 7:**
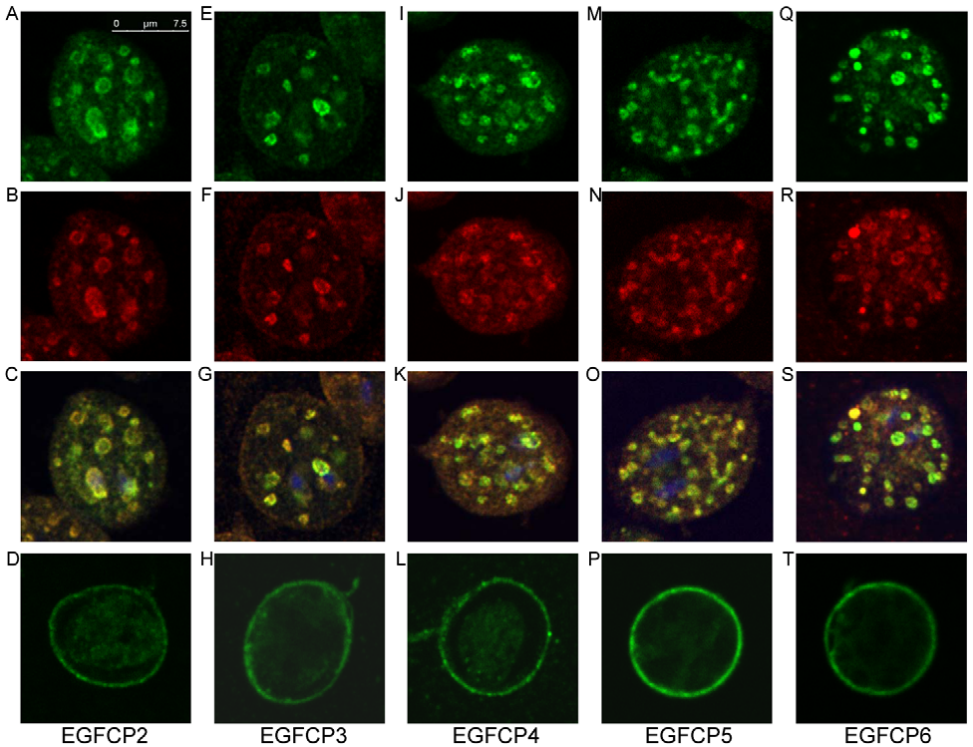
Localization of EGFCP2–6. The pPEGFCP2–6 stable transfectants were cultured in encystation medium, harvested at 24 h (Enc), and then subjected to immunofluorescence analysis using anti-HA antibody and anti-CWP1 antibody for detection. Panels A, E, I, M, and Q respectively show that HA-tagged EGFCP2, EGFCP3, EGFCP4, EGFCP5, and EGFCP6 localize to the ESVs in encysting trophozoites. Panels B, F, J, N, and R show that CWP1 localizes to the ESVs in these respective cells. Panels C, G, K, O, and S are the merged images of the DAPI staining and images of A and B, E and F, I and J, M and N, or Q and R, respectively. Panels D, H, L, P, and T respectively show that HA-tagged EGFCP2, EGFCP3, EGFCP4, EGFCP5, and EGFCP6 localize to the cyst wall and weakly to the cell body in cysts.

Amino acid sequences with similarity to that of EGFCP1 were found in five other open reading frames including open reading frames 113038 (EGFCP2), 114815 (EGFCP3), 16477 (EGFCP4), 16322 (EGFCP5) and 8687 (EGFCP6) (Fig. 1B). RT-PCR and quantitative real time PCR analysis of total RNA showed that the native transcripts of these EGFCPs were present in vegetative cells and decreased significantly in 24 h encysting cells (Fig. 6A). To further understand the function of these putative EGFCPs, we prepared construct pPEGFCP2 (or pPEGFCP3, pPEGFCP4, pPEGFCP5, pPEGFCP6) in which the *egfcp2* (or *egfcp3*, *egfcp4*, *egfcp5*, *egfcp6*) gene is controlled by its own promoter and contains an HA epitope tag at its C terminus (Fig. 6B) and stably transfected it into *G. lamblia*. A ∼70-kDa protein was detected (Fig. 6C), which is slightly larger than the predicted ∼63.0-kDa molecular mass of EGFCP3 (or EGFCP4, EGFCP5, EGFCP6) with the HA tag (∼1 kDa). The protein levels of the HA-tagged EGFCP3 (or EGFCP4, EGFCP5, EGFCP6) increased significantly during encystation (Fig. 6C). When the samples are not reduced, the HA-tagged EGFCP3 (or EGFCP4, EGFCP5, EGFCP6) was expressed as a smear with a molecular mass ranging above ∼50 kDa band, indicating the formation of larger complexes through intermolecular disulfide linkages (Fig. 6D). The HA-tagged EGFCP2 protein was not detected in either vegetative or encysting cells in Western blots (data not shown), suggesting that the expression of the *egfcp2* gene under its own promoter is very low. Using the more sensitive immunofluorescence assay, we were able to detect an up-regulation of the expression of the HA-tagged EGFCP2 under the control of its own promoter during encystation (Fig. 7A and see below).

### Overexpression of EGFCP2–6 increased the levels of cyst formation

We further investigated the effect of EGFCP2 (or EGFCP3, EGFCP4, EGFCP5, EGFCP6) on cyst formation. We found that the cyst number in the EGFCP2 (or EGFCP3, EGFCP4, EGFCP5, EGFCP6) overexpressing cell line increased by ∼1.3-2.0-fold (*p*<0.05) relative to the control cell line which expresses only the puromycin selection marker (5′▵5N-Pac) (Figs. 5A and 6E), indicating that the overexpressed EGFCP2 (or EGFCP3, EGFCP4, EGFCP5, EGFCP6) can increase the cyst formation.

### Release of EGFCP5 into medium during encystation

We further tried to understand whether EGFCP2–6 can be released into medium. The EGFCP2–6 overexpressing cell lines (pPTEGFCP2–6) were cultured in growth or encystation medium for 24 h. Both culture media were then immunoprecipitated with anti-HA antibody and then subjected to Western blot analysis. Intact EGFCP5 (a ∼70-kDa band, which is slightly larger than the predicted ∼63.1-kDa molecular mass of EGFCP5 with ∼1 kDa HA tag) was detected in the encystation medium (Fig. 6F), whereas there was no evidence for EGFCP5 release into the normal growth medium (Fig. 6F). We did not detect any released EGFCP2 (or EGFCP3, EGFCP4, EGFCP6) in the EGFCP2 (or EGFCP3, EGFCP4, EGFCP6) overexpressing cell line (data not shown). The results suggest that EGFCP5 can be released into medium during encystation.

### Localization of EGFCP2–6

Immunofluorescence assays showed that the HA-tagged EGFCP2–6 were localized to the ER in vegetative trophozoites (data not shown). During encystation, the HA-tagged EGFCP2–6 were localized to the ESVs in encysting trophozoites (Figs. 7A, E, I, M, Q). CWP1 was also colocalized with the EGFCP2–6 (Figs. 7B, C, F, G, J, K, N, O, R, S). The EGFCP2–6 were also localized to the ER and some big vesicles in a few positive encysting cells (data not shown). In the cyst stage, HA-tagged EGFCP2–6 were localized to the cyst wall plus the cell body (Figs. 7D, H, L, P, T). However, CWP1 was localized in the cyst wall in the respective cysts (data not shown). The number of positively stained cells detected in vegetative trophozoites was small (∼2% for EGFCP2, ∼15% for EGFCP3, ∼5% for EGFCP4, ∼20% for EGFCP5, ∼2% for EGFCP6) and increased (∼10% for EGFCP2, ∼50% for EGFCP3, ∼20% for EGFCP4, ∼80% for EGFCP5, ∼10% for EGFCP6) during encystation.

## Discussion

The unicellular *G. lamblia* can form a resistant cyst wall composed of proteins and polysaccharides to protect it during infection [4], [5]. To date, only a few of cyst proteins have been identified, including three CWPs and one HCNCp [7]–[9], [16]. These proteins are highly expressed during encystation and are mainly targeted to the cyst wall. In this study, we have identified a new cyst protein (EGFCP1) with nine EGF repeats by screening of the *G. lamblia* genome data base. EGFCP1 protein (but not transcript) was expressed at higher levels during encystation and it formed high molecular weight disulfide bonded complexes that were detected in non-reducing gels. We also found that EGFCP1 was localized to the ER during vegetative growth and to the ESVs of encysting trophozoites (it was also localized to the ER and some big vesicles in a few positive encysting cells, Fig. 4G) and to the cyst wall and cell body of cysts. In addition, overexpression of EGFCP1 induced the cyst formation and deletion of signal peptide sequence prevented targeting of EGFCP1 to the ESVs, and possibly to the cyst wall. This deletion also reduced the EGFCP1 protein levels and cyst formation, suggesting that a released form of EGFCP1 may help mediate the cyst wall synthesis.

Interestingly, CWP1–3, HCNCp and EGFCP1 all have signal peptides, suggesting that they can enter the secretory pathway to target to the ESVs and cyst wall. CWP1–3 are cysteine rich but the percentage is ∼5.7–7.5%, not as high as HCNCp and EGFCP1 (∼14%). CWP1, CWP3, HCNCp and EGFCP1 have acidic pI (3.59–4.72), but CWP2 has a pI of 8.13. HCNCp has an integral membrane domain, but CWP1–3 and EGFCP1 have no transmembrane domains. The CWP1–3, HCNCp, and EGFCP1 protein levels increased greatly during encystation and they could target to the ESVs of encysting trophozoites [12], [13]. It has also been shown that the levels of EGFCP1 increased by ∼3 fold during encystation according to proteomics data [43]. CWP1–3 were transported exclusively to the wall of water-resistant cysts [7]–[9]. However, HCNCp and EGFCP1 were transported to the cyst wall and cell body of cysts, suggesting that they share the same protein trafficking pathway. Like CWPs, EGFCP1 could also form disulfide bonded heterodimers and oligomers [7]–[9]. Formation of intramolecular or intermolecular disulfide bonds between the cysteines could be critical for heterooligomer formation between these cyst wall proteins in the ESVs and cyst wall [9], [14], as treatment of live encysting cells with DTT prevented the formation of disulfide bonds, ESVs and cyst wall [13], [15].

The EGF repeats of EGFCP1 are similar to those of the tenascin family of extracellular matrix glycoproteins [21]. The highly expressed tenascin-C in tumor stroma can stimulate tumor cell proliferation by stimulating signal transduction pathways [21]. EGF repeats have been found in hundreds of extracellular proteins and extracellular domains of membrane-bound proteins in humans [22], [36], [37]. A single EGF-like repeat contains ∼40 amino acids with six positionally conserved cysteines forming 3 disulfide bonds with a 1–3, 2–4, 5–6 pattern [22]. EGF-like repeats have been found in secreted proteins or cell surface proteins that function as extracellular matrix glycoproteins, cytokines, sensors or receptors [21], [22], [36], [37]. They are involved in many different physiological functions, including extracellular matrix remodeling, coagulation, fibrinolysis, cell adhesion, cell mobility, development, cell proliferation and differentiation, and cell cycle progression [21], [22], [36], [37]. However, the functions of the EGF-like repeats in these proteins are largely unknown and some of them are known to be important for protein-protein interaction or cell adhesion [22], [36], [37]. It has also been shown that the EGF-like repeats in tenascin-C can modulate cell adhesion [44] and can act directly as ligands for EGF receptor to activate EGF receptor signaling and induce cell mitosis [45]. In addition, it has been shown that a microneme protein with EGF repeat is functional for host cell receptor recognition when secreted onto the surface of *Toxoplasma gondii* [46]. Giardial cyst wall is a resistant extracellular wall that helps cysts survive in the hostile environment and infect a new host [2]. It is possible that giardial proteins with EGF repeats may also be extracellular proteins or membrane-bound proteins and that they may use the EGF repeats to modulate cell adhesion or induce signaling. In additional to giardial EGFCP1, some HCMps (EGF group of HCMps) also have EGF repeats [16]. HCNCp also has nine EGF repeats as predicted by SMART analysis (http://smart.embl-heidelberg.de) (data not shown). The similar localization of EGFCP1 and HCNCp in the ESVs, cyst wall and cell body of cysts [16] suggests that they may share the same novel protein trafficking pathway and they may have similar functions, including providing structural support of cyst wall. It has been proposed that the cell body is the precursor of the excyzoite and the localization of HCNCp and EGFCP1 in the cyst cell body suggests that they may have a function during or after excystation [16]. It is also possible that EGFCP1 and HCNCp may induce signal transduction during or after excystation when they are on the cyst wall. It is possible that EGFCP1 may act as a ligand to induce encystation-specific signal transduction.

EGFCP1 has two putative TIL cysteine rich domains (Pfam: PF01826) (Fig. 1B). One is overlapped with an EGF repeat with all conserved cysteines (Fig. 1B). TIL domains have been found in some protease inhibitors. For example, Chymotrypsin/elastase inhibitors with TIL domains have been found in *Ascaris suum* to protect the parasites from host digestive enzymes [47]. The two TIL domains of EGFCP1 are not typical because only 9 conserved cysteines were found (one more cysteine can be found outside of the TIL domain) (Fig. S4). We were unable to detect any inhibition of trypsin and chymotrypsin activity by recombinant EGFCP1 despite using a variety of conditions (data not shown), suggesting that the TIL domains of EGFCP1 are not complete and not functional.

To date, only COWP1 and COWP8 have been localized to the oocyst wall which are of interest [17], [18]. We chose the type I domain to do the search because every COWP has type I domain but not type II domain. EGFCP1 was identified by screening of the *G. lamblia* genome data base using the type I domain of COWP8 as a query sequence. This search detected three other open reading frames, including 114161, 14331, and 17328 (data not shown). Open reading frames 14331 and 17328 were annotated as VSP and HCMp, respectively. Open reading frame 114161 was described as a cysteine-rich protein with a C-terminal transmembrane region [16]. It was not classified as HCMp because it did not match the characteristics of ≥20 CxC and/or CxxC [16].

Five other open reading frames were identified by searching the *G. lamblia* genome data base with the amino acid sequences of the EGFCP1, including open reading frames 113038 (EGFCP2), 114815 (EGFCP3), 16477 (EGFCP4), 16322 (EGFCP5) and 8687 (EGFCP6). They had high expectation values (from 6.6e^−149^ to 5.0e^−64^) to EGFCP1. We found that these putative EGFCPs have similar characteristics with EGFCP1: i) they were expressed at higher levels during encystation (except that the expression of EGFCP2 was too low to be detected); ii) they formed high molecular weight disulfide bonded complexes that were detected in non-reducing gels (except that the expression of EGFCP2 was too low to be detected); iii) they were localized to the ER during vegetative growth and partly to the ESVs of encysting trophozoites and to the cyst wall and cell body of cysts; iv) Overexpression of these five putative EGFCPs induced the cyst formation. Like EGFCP1, EGFCP5 can also be released into the medium during encystation. We did not detect the release of EGFCP2, 3, 4, 6 into the medium, possibly because they are too little to be detected. All the six putative EGFCPs (including EGFCP1) have acidic pIs, signal peptides (Signal P prediction) [34], >10% cysteines and >8 EGF repeats, but they have no transmembrane domains (Fig. 2). Interestingly, sixty-seven cysteines of these putative EGFCPs are positionally conserved. We also detected 44 other open reading frames with high expectation values (from 4.8e^−73^ to 4.1e^−10^) to EGFCP1 (data not shown). Among these open reading frames, two have been annotated as VSPs. One have been annotated as HCNCp. Twenty-four have been annotated as HCMps. Six have been annotated as cysteine-rich proteins but not classified as HCMps (they are too short and/or have <20 CxC and/or CxxC) [16]. Five have transmembrane domains as predicted by TMHMM (http://www.cbs.dtu.dk/services/TMHMM/) (data not shown). Finally, there are six open reading frames without transmembrane domains as predicted by TMHMM (data not shown and Fig. S6). They also have some characteristics of EGFCPs (Fig. S6). They are cysteine-rich proteins with variable sizes and numbers of EGF repeats (257–1093 amino acids and 2–11 EGF repeats) (Fig. S6). However, only five and four of them have signal peptides (Signal P prediction) [34] and acidic pIs, respectively (Fig. S6). We were not able to include these open reading frames into the list of EGFCPs (in Figs. 1B and 2) because of their variable sizes and numbers of EGF repeats. Further studies will be required to elucidate the functions of these putative EGFCPs.

Signal peptide is important for protein secretion and its deletion may result in loss of protein function. It has been shown that overexpression of the human hyaluronan and proteoglycan linked protein 1 can increase tumorigenicity and deletion of the signal peptide can decrease its tumorigenic activity [48]. The results of our study revealed that overexpression of EGFCP1 induced the cyst formation and that deletion of the N-terminal signal peptide (the EGFCP1Δsp mutant) led to a decrease of cyst formation. Similarly, we found that overexpression of CWP3 resulted in an increase of cyst formation and that deletion of the N-terminal signal peptide of CWP3 resulted in a decrease of cyst formation (Sun et al., unpublished data). The EGFCP1Δsp protein was present in cytosol and did not enter the secretory pathway to reach the ESVs, and possibly cyst wall. In addition, the protein levels of the EGFCP1Δsp in the pPTEGFCP1Δsp cell line were very low relative to that in the pPTEGFCP1 cell line, indicating that N-terminal coding region for signal peptide was required for the EGFCP1 protein translation or stability. The different expression levels could be not due to different plasmid copy numbers in the pTEGFCP1Δsp and pTEGFCP1 cell lines, because similar plasmid copy numbers were found in the these cell lines (data not shown). This indicates that N-terminal coding region for signal peptide was required for the EGFCP1 protein translation or stability. As shown in RT-PCR and quantitative real time PCR, the decreased levels of EGFCP1Δsp protein were due, at least in part, to a decrease of steady state mRNA levels, suggesting that the signal peptide coding region may interact with regulatory protein factors that may play a role in regulation of transcription initiation or RNA stability.

There is a reverse correlation between the steady state mRNA and protein levels of the EGFCPs. During encystation, the endogenous protein levels of the EGFCP1 increased, but the endogenous mRNA levels of the EGFCP1 decreased. We also found that the protein levels of the epitope-tagged EGFCPs increased during encystation, but the mRNA levels of the tagged EGFCPs decreased, even when the encystation-reduced *α2-tubulin* promoter were used to drive the EGFCP1 expression. Further studies will be required to elucidate whether the increased protein levels of the EGFCPs could be due to an increase in their translation rate or protein half life during encystation. It is also possible that protein turnover may be decreased during encystation.

Our findings provide new insight into distinct function of cysteine-rich EGF-repeats containing cyst proteins in differentiation of *G. lamblia* trophozoites into cysts. Our findings also lead to greater understanding of parasite cyst wall, which is more complex than expected, and provide a model to investigate the mechanism of extracellular matrix secretion and assembly.

## Supporting Information

Figure S1 Schematic representation of plasmid construction. For constructing pPTEGFCP1, a PCR with oligonucleotides TUBNF (GGCGGCTAGCCGCAGACGCATGA) and TUBBR (GGCGGGATCCTTTTATTTCCGCCCGTCCAG) generated a 0.3-kb product that was digested with *Nhe*I and *Bam*HI. Another PCR with primers egfcp1BF (GGCGGAGATCTATGATAGCCGCGGCCTTTCTC) and egfcp1MR (GGCGACGCGTCACACATCTACCATCGC) generated a 1.7-kb PCR product that was digested with *Bgl*II and *Mlu*I and cloned into *Nhe*I/*Mlu*I digested pPop2NHA [27] with the 0.3-kb *Nhe*I/*Bam*HI fragment. The resulting plasmid, pPTEGFCP1, contained the *egfcp1* gene controlled by *α2-tubulin* promoter with an HA tag fused at its C-terminus. For constructing pNEGFCP1, we integrated the AU1 tag sequence into the pNLop2-1 vector [28]. Complementary oligonucleotides AUF (CTAGCAAAAACGCGTGATACGTATCGATACATCTAAG) and AUR (AATTCTTAGATGTATCGATACGTATCACGCGTTTTTG) were phosphorylated and annealed. The double-strand DNA fragment was ligated into *Nhe*I/*Eco*RI-digested and dephosphorylated pNLop2-1 to produce pNAU. The *egfcp1* gene and its 360-bp 5′-flanking region was amplified with oligonucleotides egfcp1NF (GGCGGCTAGCTCAACAGAGGAGAACGA) and egfcp1MR, digested with *Nhe*I/*Mlu*I, and ligated in place of the *Nhe*I/*Mlu*I-excised luciferase gene in pNAU. The resulting plasmid, pNEGFCP1, contained the *egfcp1* gene controlled by its own promoter with an AU1 tag fused at its C-terminus. For constructing pPTEGFCP1Δsp, a PCR with oligonucleotides egfcp1dBF (GGGCGAGATCTATGGATGGCGAGGTGGATGTAAAC), egfcp1MR generated a 1.5-kb product was digested with BglII and MluI, and cloned into *Bam*HI/*Mlu*I digested pPTEGFCP1. The resulting plasmid, pPTEGFCP1Δsp, contains an egfcp1 gene lacking the coding sequence for the predicted signal peptide sequence (nucleotides 4–48, residues 2–16). For constructing pPEGFCP2 (or pPEGFCP4, pPEGFCP5, or pEGFCP6), a PCR with oligonucleotides EGFCP2NF (GGGCGGCTAGCGAGGAGCTCTCTGCGAAAGAC) and EGFCP2MR (GGGCGACGCGTATTGCCATAGAAACAAGCTCC) (or EGFCP4NF (GGGCGGCTAGCTGCTGGTTTGAAGGAGGCGCT) and EGFCP4MR (GGGCGACGCGTCGCTGCTCTTTCACAATATCC), EGFCP5NF (GGGCGGCTAGCTGGTCTTAAAGCGGCTGATGG) and EGFCP5MR (GGGCGACGCGTAAGTGTTTTTACATTACAGTG), EGFCP6NF (GGGCGGCTAGCTGACCTTGGGGGCAACGGGCG) and EGFCP6MR (GGGCGACGCGTCTGGGAGCTGCTGACTGCACA)) generated a 1.7-kb product was digested with *Nhe*I and *Mlu*I, and cloned into *Nhe*I/*Mlu*I digested pPTEGFCP1. The resulting plasmid, pPEGFCP2, pPEGFCP4, pPEGFCP5, or pPEGFCP6, contains the *egfcp2, egfcp4, egfcp5*, or *egfcp6* gene controlled by its own promoter with an HA tag fused at its C-terminus. For constructing pPEGFCP3, a PCR with oligonucleotides EGFCP3NF (GGGCGGCTAGCTTAGAGTAAATTTAAGGGACT) and EGFCP3XR (GGGCGTCTAGACTATACCCATACGATGTTCCAGATTACGCTATCGCTATAGA AACAGGCTCC) generated a 1.7-kb product was digested with *Nhe*I and *Xba*I, and cloned into *Nhe*I/*Xba*I digested pPop2N [27]. The resulting plasmid, pPEGFCP3, contains an *egfcp3* gene controlled by its own promoter with an HA tag fused at its C-terminus.
(0.03 MB PDF)

Figure S2 Comparison of TSA417, HCNCp and EGFCP1. (A) Summary of characteristics of TSA417, HCNCp and EGFCP1. TSA417 is a representative of VSPs. (B) Comparison of cysteines of EGFCPs. The number of cysteine-containing motifs from Cx0C to Cx20C is shown. The number zero is not shown.
(0.01 MB PDF)

Figure S3 Alignment of the amino acid sequences of the EGF or EGF-like repeats of EGFCP1. The EGF or EGF-like repeats predicted by SMART analysis (http://smart.embl-heidelberg.de) in Fig. 1B were compared respectively. Amino acids that are similar or identical to the consensus according to Clustal W 1.83 [49] are indicated in gray or black. Six positionally conserved cysteines are shown with black triangles. Consensus sequence of EGF-like repeats is shown below (Campbell LD, Bork, P. (1993) Epidermal growth factor-like modules. Current Opinions in Structural Biology. 3: 385–392.).
(0.06 MB PDF)

Figure S4 Alignment of the amino acid sequences of the two putative TIL domains of EGFCP1 and *Ancylostoma caninum* anti-coagulant precursors (AcSP6 and AcASP5), *A. ceylanicum* Ascaris-type serine protease inhibitor (Acl), and *Apis mellifera* (honeybee) chymotrypsin inhibitor (AMCI) (Accession numbers AAC47081, AAC47082, AAD51336, and P56682, respectively). All alignments were carried out using Clustal W 1.83 [49]. Amino acids that are similar or identical to the consensus are indicated in gray or black. The TIL domain has 10 positionally conserved cysteine residues forming 5 disulfide bonds [41]. Eight positionally conserved cysteines are shown with black triangles. Two positionally conserved cysteines of these proteins except those in the two TIL domains of EGFCP1 are indicated with gray triangles. Cysteines that are not positionally conserved in the two TIL domains of EGFCP1 are indicated with gray arrows. Each of the two TIL domains of EGFCP1 has 9 cysteines and one more cysteine is located in the N-terminal extended sequence (see inserted sequence).
(0.03 MB PDF)

Figure S5 Coomassie Blue staining of purified EGFCP1. Recombinant EGFCP1 protein was purified from *E. coli* using nickel affinity chromatography under native conditions. Purified EGFCP1 protein was analyzed by SDS-PAGE and Coomassie Blue staining. (B) EGFCP1 protein levels in different stages. The wild-type non-transfected WB cells were cultured in growth (Veg) or encystation medium (Enc) for 2, 5, 9, and 24 h and then subjected to SDS-PAGE and Western blot. The blot was probed by anti-EGFCP1 antibody. A representative full-length result is shown (also see Fig. 3B). (C) Detection of EGFCP1Δsp. This figure is a longer exposure of anti-HA part of the Fig. 8D to show the presence of HA-tagged EGFCP1Δsp.
(0.11 MB PDF)

Figure S6 Six open reading frames with some characteristics of EGFCPs. Amino acid sequences with similarity to that of EGFCP1 were found in six other open reading frames including 92495, 16833, 14573, 10330, 113268 and 103983.The number and location of the EGF or EGF-like repeats in EGFCPs are predicted by SMART analysis (http://smart.embl-heidelberg.de). Two to eleven EGF or EGF-like repeats are present in these open reading frames. Thirty-four to one hundred and forty-six cysteines are present in these open reading frames. They have no transmembrane domains as predicted by TMHMM (http://www.cbs.dtu.dk/services/TMHMM/). Some have acidic pIs and signal peptides (black boxes) as predicted by Signal P [34].
(0.05 MB PDF)

Table S1 Primers used for semi-quantitative RT-PCR and quantitative real time PCR.
(0.01 MB PDF)
