# Supplementary material for: A Novel Family of Cyst Proteins with Epidermal Growth Factor Repeats in Giardia lamblia
Source: PLoS Negl Trop Dis. 2010 May 11;4(5):e677. doi: 10.1371/journal.pntd.0000677 (PMC2867935; doi:10.1371/journal.pntd.0000677)
Supplement: Figure S1 — Schematic representation of plasmid construction. For constructing pPTEGFCP1, a PCR with oligonucleotides TUBNF (GGCGGCTAGCCGCAGACGCATGA) and TUBBR (GGCGGGATCCTTTTATTTCCGCCCGTCCAG) generated a 0.3-kb product that was digested with NheI and BamHI. Another PCR with primers egfcp1BF (GGCGGAGATCTATGATAGCCGCGGCCTTTCTC) and egfcp1MR (GGCGACGCGTCACACATCTACCATCGC) generated a 1.7-kb PCR product that was digested with BglII and MluI and cloned into NheI/MluI digested pPop2NHA [27] with the 0.3-kb NheI/BamHI fragment. The resulting plasmid, pPTEGFCP1, contained the egfcp1 gene controlled by α2-tubulin promoter with an HA tag fused at its C-terminus. For constructing pNEGFCP1, we integrated the AU1 tag sequence into the pNLop2-1 vector [28]. Complementary oligonucleotides AUF (CTAGCAAAAACGCGTGATACGTATCGATACATCTAAG) and AUR (AATTCTTAGATGTATCGATACGTATCACGCGTTTTTG) were phosphorylated and annealed. The double-strand DNA fragment was ligated into NheI/EcoRI-digested and dephosphorylated pNLop2-1 to produce pNAU. The egfcp1 gene and its 360-bp 5′-flanking region was amplified with oligonucleotides egfcp1NF (GGCGGCTAGCTCAACAGAGGAGAACGA) and egfcp1MR, digested with NheI/MluI, and ligated in place of the NheI/MluI-excised luciferase gene in pNAU. The resulting plasmid, pNEGFCP1, contained the egfcp1 gene controlled by its own promoter with an AU1 tag fused at its C-terminus. For constructing pPTEGFCP1Δsp, a PCR with oligonucleotides egfcp1dBF (GGGCGAGATCTATGGATGGCGAGGTGGATGTAAAC), egfcp1MR generated a 1.5-kb product was digested with BglII and MluI, and cloned into BamHI/MluI digested pPTEGFCP1. The resulting plasmid, pPTEGFCP1Δsp, contains an egfcp1 gene lacking the coding sequence for the predicted signal peptide sequence (nucleotides 4–48, residues 2–16). For constructing pPEGFCP2 (or pPEGFCP4, pPEGFCP5, or pEGFCP6), a PCR with oligonucleotides EGFCP2NF (GGGCGGCTAGCGAGGAGCTCTCTGCGAAAGAC) and EGFCP2MR (GGGCGACGCGTATTGCCATAGAAACAAGCTCC) (or EGFCP4NF (GGGCGGCTAGCTGCTGGTTTGAAGGAGGCGCT) [file pntd.0000677.s001.pdf]

Figure S1

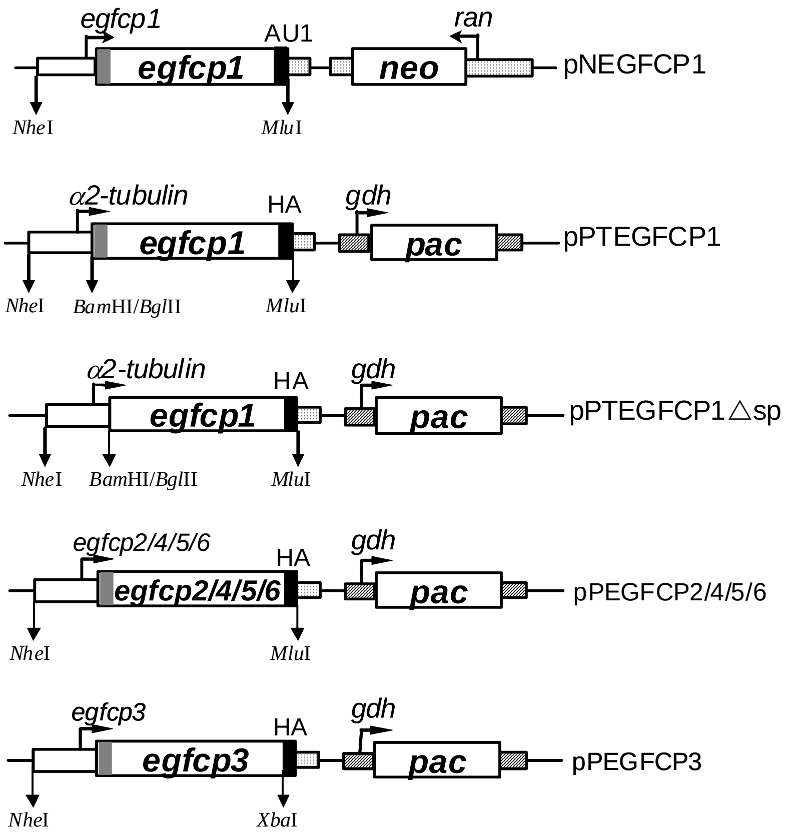

Fig. S1. Schematic representation of plasmid construction. For constructing pPTEGFCP1, a PCR with oligonucleotides TUBNF (GGCGGCTAGCCGCAGACGCATGA) and TUBBR (GGCGGGATCCTTTTATTTCCGCCCGTCCAG) generated a 0.3-kb product that was digested with *Nhe*I and *Bam*HI. Another PCR with primers egfcp1BF (GGCGGAGATCTATGATAGCCGCGGCCTTTCTC) and egfcp1MR (GGCGACGCGTCACACATCTACCATCGC) generated a 1.7-kb PCR product that was digested with *Bgl*II and *Mlu*I and cloned into *Nhe*I/*Mlu*I digested pPop2NHA [27] with the 0.3-kb *Nhe*I/*Bam*HI fragment. The resulting plasmid, pPTEGFCP1, contained the *egfcp1* gene controlled by *α2-tubulin* promoter with an HA tag fused at its C-terminus. For constructing pNEGFCP1, we integrated the AU1 tag sequence into the pNLop2-1 vector [28]. Complementary oligonucleotides AUF (CTAGCAAAAACGCGTGATACGTATCGATACATCTAAG) and AUR (AATTCTTAGATGTATCGATACGTATCACGCGTTTTTG) were phosphorylated and annealed. The double-strand DNA fragment was ligated into *Nhe*I/*Eco*RI-digested and dephosphorylated pNLop2-1 to produce pNAU. The *egfcp1* gene and its 360-bp

5’-flanking region was amplified with oligonucleotides egfcp1NF (GGCGGCTAGCTCAACAGAGGAGAACGA) and egfcp1MR, digested with *Nhe*I/*Mlu*I, and ligated in place of the *Nhe*I/*Mlu*I-excised luciferase gene in pNAU. The resulting plasmid, pNEGFCP1, contained the *egfcp1* gene controlled by its own promoter with an AU1 tag fused at its C-terminus. For constructing pPTEGFCP1△sp, a PCR with oligonucleotides egfcp1dBF (GGGCGAGATCTATGGATGGCGAGGTGGATGTAAAC), egfcp1MR generated a 1.5-kb product was digested with *Bgl*II and *Mlu*I, and cloned into *Bam*HI/*Mlu*I digested pPTEGFCP1. The resulting plasmid, pPTEGFCP1△sp, contains an *egfcp1* gene lacking the coding sequence for the predicted signal peptide sequence (nucleotides 4-48, residues 2-16). For constructing pPEGFCP2 (or pPEGFCP4, pPEGFCP5, or pEGFCP6), a PCR with oligonucleotides EGFCP2NF (GGGCGGCTAGCGAGGAGCTCTCTGCGAAAGAC) and EGFCP2MR (GGGCGACGCGTATTGCCATAGAAACAAGCTCC) (or EGFCP4NF (GGGCGGCTAGCTGCTGGTTTGAAGGAGGCGCT) and EGFCP4MR (GGGCGACGCGTCGCTGCTCTTTCACAATATCC), EGFCP5NF (GGGCGGCTAGCTGGTCTTAAAGCGGCTGATGG) and EGFCP5MR (GGGCGACGCGTAAGTGTTTTTACATTACAGTG), EGFCP6NF (GGGCGGCTAGCTGACCTTGGGGGCAACGGGCG) and EGFCP6MR (GGGCGACGCGTCTGGGAGCTGCTGACTGCACA)) generated a 1.7-kb product was digested with *Nhe*I and *Mlu*I, and cloned into *Nhe*I/*Mlu*I digested pPTEGFCP1. The resulting plasmid, pPEGFCP2, pPEGFCP4, pPEGFCP5, or pPEGFCP6, contains the *egfcp2*, *egfcp4*, *egfcp5*, or *egfcp6* gene controlled by its own promoter with an HA tag fused at its C-terminus. For constructing pPEGFCP3, a PCR with oligonucleotides EGFCP3NF (GGGCGGCTAGCTTAGAGTAAATTTAAGGGACT) and EGFCP3XR (GGGCGTCTAGACTATACCCATACGATGTTCCAGATTACGCTATCGCTATAGAAACAGGCTCC) generated a 1.7-kb product was digested with *Nhe*I and *Xba*I, and cloned into *Nhe*I/*Xba*I digested pPop2N [27]. The resulting plasmid, pPEGFCP3, contains an *egfcp3* gene controlled by its own promoter with an HA tag fused at its C-terminus.
