# Supplementary material for: A Novel Family of Cyst Proteins with Epidermal Growth Factor Repeats in Giardia lamblia
Source: PLoS Negl Trop Dis. 2010 May 11;4(5):e677. doi: 10.1371/journal.pntd.0000677 (PMC2867935; doi:10.1371/journal.pntd.0000677)
Supplement: Figure S2 — Comparison of TSA417, HCNCp and EGFCP1. (A) Summary of characteristics of TSA417, HCNCp and EGFCP1. TSA417 is a representative of VSPs. (B) Comparison of cysteines of EGFCPs. The number of cysteine-containing motifs from Cx0C to Cx20C is shown. The number zero is not shown. (0.01 MB PDF) [file pntd.0000677.s002.pdf]

Figure S2

A

	TSA 417	HCNCp	EGFCP1
Molecular weight	72.5	169.4	61.8
% of Cys	12	14	14.2
pI	4.7	4.72	4.12
Signal peptide	+	+	+
Number of transmembrane domains	1	1	0
C-terminus	CRGKA	CCRRSKAV	CRDGRCV

B

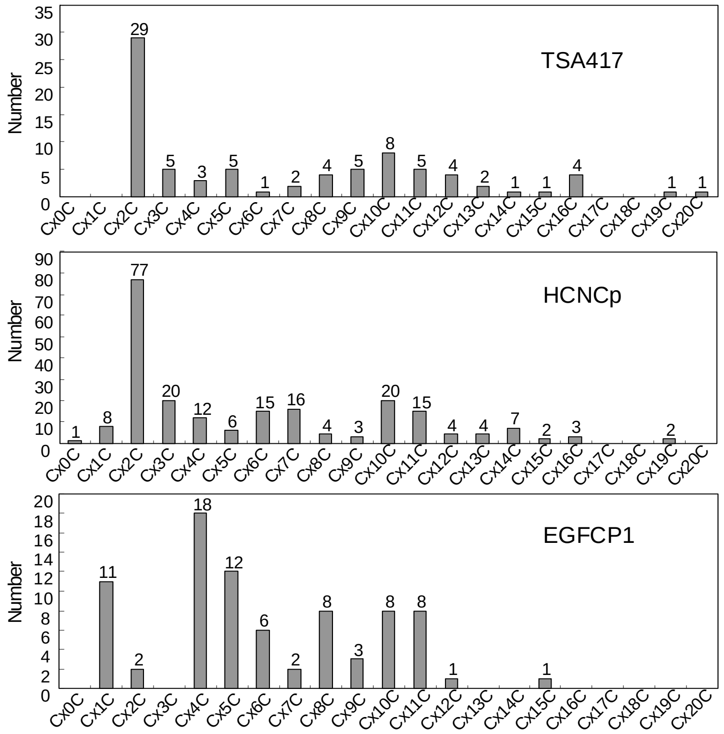

Fig. S2. Comparison of TSA417, HCNCp and EGFCP1. (A) Summary of characteristics of TSA417, HCNCp and EGFCP1. TSA417 is a representative of VSPs. (B) Comparison of cysteines of EGFCPs. The number of cysteine-containing motifs from Cx0C to Cx20C is shown. The number zero is not shown.
