# Supplementary material for: A Novel Family of Cyst Proteins with Epidermal Growth Factor Repeats in Giardia lamblia
Source: PLoS Negl Trop Dis. 2010 May 11;4(5):e677. doi: 10.1371/journal.pntd.0000677 (PMC2867935; doi:10.1371/journal.pntd.0000677)
Supplement: Figure S3 — Alignment of the amino acid sequences of the EGF or EGF-like repeats of EGFCP1. The EGF or EGF-like repeats predicted by SMART analysis (http://smart.embl-heidelberg.de) in Fig. 1B were compared respectively. Amino acids that are similar or identical to the consensus according to Clustal W 1.83 [49] are indicated in gray or black. Six positionally conserved cysteines are shown with black triangles. Consensus sequence of EGF-like repeats is shown below (Campbell LD, Bork, P. (1993) Epidermal growth factor-like modules. Current Opinions in Structural Biology. 3: 385–392.). (0.06 MB PDF) [file pntd.0000677.s003.pdf]

Figure S3

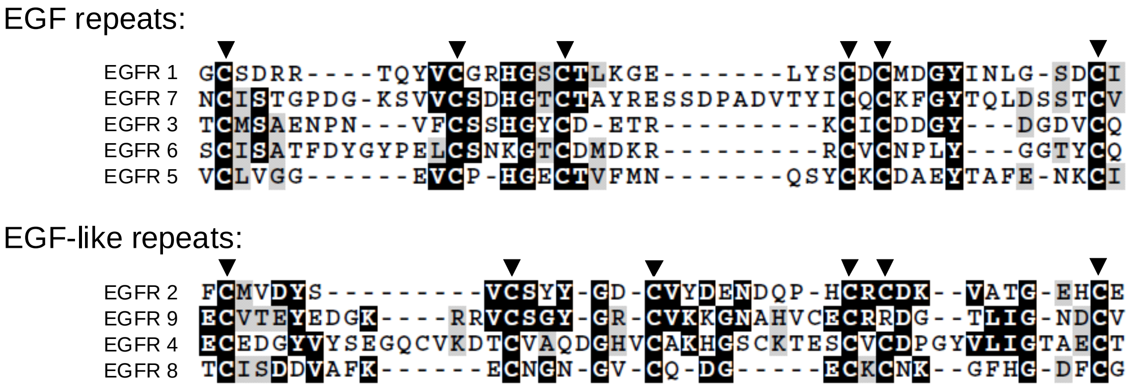

Consensus EGF-like repeats: $CX_{2\text{-}7}CX_{1\text{-}4}GXCX_{1\text{-}17}CXCXXG(Y/W/F)X_{1\text{-}6}GXXCX$

Fig. S3. Alignment of the amino acid sequences of the EGF or EGF-like repeats of EGFCP1. The EGF or EGF-like repeats predicted by SMART analysis (http://smart.embl-heidelberg.de) in Fig. 1B were compared respectively. Amino acids that are similar or identical to the consensus according to Clustal W 1.83 [49] are indicated in gray or black. Six positionally conserved cysteines are shown with black triangles. Consensus sequence of EGF-like repeats is shown below (Campbell LD, Bork, P. (1993) Epidermal growth factor-like modules. Current Opinions in Structural Biology. 3: 385-392.).
