# Supplementary material for: A Novel Family of Cyst Proteins with Epidermal Growth Factor Repeats in Giardia lamblia
Source: PLoS Negl Trop Dis. 2010 May 11;4(5):e677. doi: 10.1371/journal.pntd.0000677 (PMC2867935; doi:10.1371/journal.pntd.0000677)
Supplement: Figure S4 — Alignment of the amino acid sequences of the two putative TIL domains of EGFCP1 and Ancylostoma caninum anti-coagulant precursors (AcSP6 and AcASP5), A. ceylanicum Ascaris-type serine protease inhibitor (Acl), and Apis mellifera (honeybee) chymotrypsin inhibitor (AMCI) (Accession numbers AAC47081, AAC47082, AAD51336, and P56682, respectively). All alignments were carried out using Clustal W 1.83 [49]. Amino acids that are similar or identical to the consensus are indicated in gray or black. The TIL domain has 10 positionally conserved cysteine residues forming 5 disulfide bonds [41]. Eight positionally conserved cysteines are shown with black triangles. Two positionally conserved cysteines of these proteins except those in the two TIL domains of EGFCP1 are indicated with gray triangles. Cysteines that are not positionally conserved in the two TIL domains of EGFCP1 are indicated with gray arrows. Each of the two TIL domains of EGFCP1 has 9 cysteines and one more cysteine is located in the N-terminal extended sequence (see inserted sequence). (0.03 MB PDF) [file pntd.0000677.s004.pdf]

Figure S4

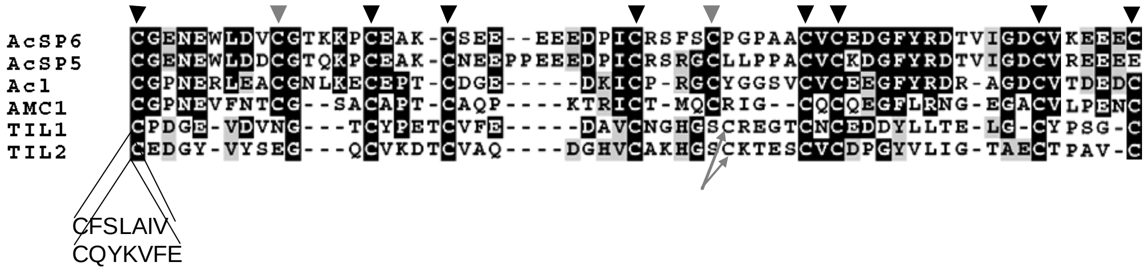

Fig. S4. Alignment of the amino acid sequences of the two putative TIL domains of EGFCP1 and *Ancylostoma caninum* anti-coagulant precursors (AcSP6 and AcASP5), *A. ceylanicum Ascaris*-type serine protease inhibitor (Acl), and *Apis mellifera* (honeybee) chymotrypsin inhibitor (AMCI) (Accession numbers AAC47081, AAC47082, AAD51336, and P56682, respectively). All alignments were carried out using Clustal W 1.83 [49]. Amino acids that are similar or identical to the consensus are indicated in gray or black. The TIL domain has 10 positionally conserved cysteine residues forming 5 disulfide bonds [41]. Eight positionally conserved cysteines are shown with black triangles. Two positionally conserved cysteines of these proteins except those in the two TIL domains of EGFCP1 are indicated with gray triangles. Cysteines that are not positionally conserved in the two TIL domains of EGFCP1 are indicated with gray arrows. Each of the two TIL domains of EGFCP1 has 9 cysteines and one more cysteine is located in the N-terminal extended sequence (see inserted sequence).
