# Supplementary material for: A Novel Family of Cyst Proteins with Epidermal Growth Factor Repeats in Giardia lamblia
Source: PLoS Negl Trop Dis. 2010 May 11;4(5):e677. doi: 10.1371/journal.pntd.0000677 (PMC2867935; doi:10.1371/journal.pntd.0000677)
Supplement: Figure S5 — Coomassie Blue staining of purified EGFCP1. Recombinant EGFCP1 protein was purified from E. coli using nickel affinity chromatography under native conditions. Purified EGFCP1 protein was analyzed by SDS-PAGE and Coomassie Blue staining. (B) EGFCP1 protein levels in different stages. The wild-type non-transfected WB cells were cultured in growth (Veg) or encystation medium (Enc) for 2, 5, 9, and 24 h and then subjected to SDS-PAGE and Western blot. The blot was probed by anti-EGFCP1 antibody. A representative full-length result is shown (also see Fig. 3B). (C) Detection of EGFCP1Δsp. This figure is a longer exposure of anti-HA part of the Fig. 8D to show the presence of HA-tagged EGFCP1Δsp. (0.11 MB PDF) [file pntd.0000677.s005.pdf]

Figure S5

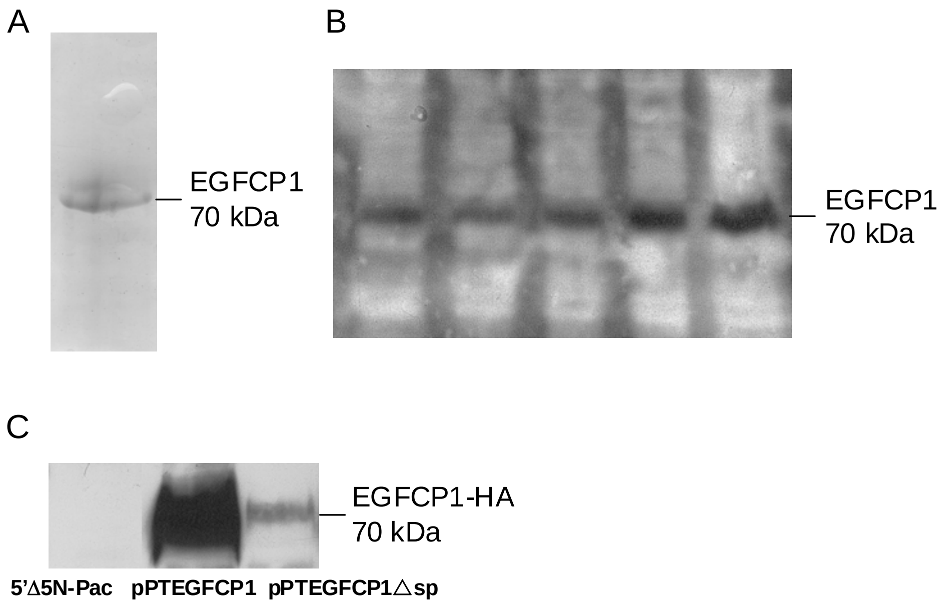

Fig. S5. (A) Coomassie Blue staining of purified EGFCP1. Recombinant EGFCP1 protein was purified from *E. coli* using nickel affinity chromatography under native conditions. Purified EGFCP1 protein was analyzed by SDS-PAGE and Coomassie Blue staining. (B) EGFCP1 protein levels in different stages. The wild-type non-transfected WB cells were cultured in growth (Veg) or encystation medium (Enc) for 2, 5, 9, and 24 h and then subjected to SDS-PAGE and Western blot. The blot was probed by anti-EGFCP1 antibody. A representative full-length result is shown (also see Fig. 3B). (C) Detection of EGFCP1△sp. This figure is a longer exposure of anti-HA part of the Fig. 8D to show the presence of HA-tagged EGFCP1△sp.
