# Supplementary material for: A Novel Family of Cyst Proteins with Epidermal Growth Factor Repeats in Giardia lamblia
Source: PLoS Negl Trop Dis. 2010 May 11;4(5):e677. doi: 10.1371/journal.pntd.0000677 (PMC2867935; doi:10.1371/journal.pntd.0000677)
Supplement: Figure S6 — Six open reading frames with some characteristics of EGFCPs. Amino acid sequences with similarity to that of EGFCP1 were found in six other open reading frames including 92495, 16833, 14573, 10330, 113268 and 103983.The number and location of the EGF or EGF-like repeats in EGFCPs are predicted by SMART analysis (http://smart.embl-heidelberg.de). Two to eleven EGF or EGF-like repeats are present in these open reading frames. Thirty-four to one hundred and forty-six cysteines are present in these open reading frames. They have no transmembrane domains as predicted by TMHMM (http://www.cbs.dtu.dk/services/TMHMM/). Some have acidic pIs and signal peptides (black boxes) as predicted by Signal P [34]. (0.05 MB PDF) [file pntd.0000677.s006.pdf]

Figure S6

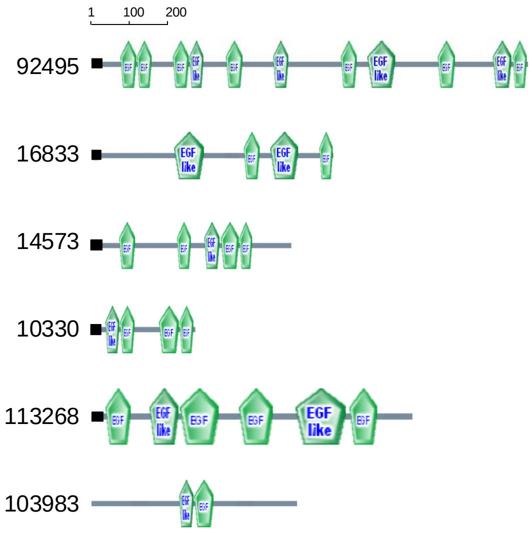

Number of EGF or EGF-like repeats	Number (percentage) of cysteines	Number of transmembrane domains	pI	Molecular weight	Amino acids
11	146 (13.4%)	0	5.19	118.8	1093
4	81 (13.4%)	0	4.63	64.7	605
5	68 (13.6%)	0	4.82	53.4	499
4	34 (13.2%)	0	4.70	28.1	257
6	48 (11.4%)	0	6.02	44.2	420
2	57 (11.1%)	0	6.23	55.0	516

Fig. S6 Six open reading frames with some characteristics of EGFCPs. Amino acid sequences with similarity to that of EGFCP1 were found in six other open reading frames including 92495, 16833, 14573, 10330, 113268 and 103983.The number and location of the EGF or EGF-like repeats in EGFCPs are predicted by SMART analysis (http://smart.embl-heidelberg.de). Two to eleven EGF or EGF-like repeats are present in these open reading frames. Thirty-four to one hundred and forty-six cysteines are present in these open reading frames. They have no transmembrane domains as predicted by TMHMM (http://www.cbs.dtu.dk/services/TMHMM/). Some have acidic pIs and signal peptides (black boxes) as predicted by Signal P [34].
