# Supplementary material for: A Novel Family of Cyst Proteins with Epidermal Growth Factor Repeats in Giardia lamblia
Source: PLoS Negl Trop Dis. 2010 May 11;4(5):e677. doi: 10.1371/journal.pntd.0000677 (PMC2867935; doi:10.1371/journal.pntd.0000677)
Supplement: Table S1 — Primers used for semi-quantitative RT-PCR and quantitative real time PCR. (0.01 MB PDF) [file pntd.0000677.s007.pdf]

Table S1. Primers used for semi-quantitative RT-PCR and quantitative real time PCR.

Name	Sequence (5' to 3')
egfcp1F	CACCATGATAGCCGCGGCCTTTCT
egfcp1R	CACACATCTACCATCGCGAC
egfcp1realF	GCTTATTGGCACCGCTGAGT
egfcp1realR	CAAACTTCTCCGCCAACAAGA
egfcp1HAR	AGCGTAATCTGGAACATCGTATGGGTA
egfcp1HAF	GCAACGCACACGTCTGTGA
egfcp13F	CAGTACAAGGTCTTCGAGTGC
egfcp13R	AAAGATGCTCTAAACGGAT
egfcp2realF	CCCCAATTCCTGTGTCACTGA
egfcp2realR	CCCAGTCCAGAGCAGAGCTT
egfcp3realF	TGGAGTGTGTGTTGCTGAGGAT
egfcp3realR	GCAGAAGGTCCCAGCGTAGA
egfcp3R	ATCGCTATAGAAACAGGCTCC
egfcp4realF	GGTGGAGAGTGCGTCAATCA
egfcp4realR	CCCCCGGAGCAAACGT
egfcp4R	TGCTGGTTTGAAGGAGGCGCT
egfcp5realF	CGTCCTCGTGTGTCACCAATAG
egfcp5realR	ATCCCGCACTGTGACATAGCT
egfcp5R	TGGTCTTAAAGCGGCTGATGG
egfcp6realF	GAAGGACCGCACTGCCAAT
egfcp6realR	CTCGCTTTGCGCATATCTCA
egfcp6R	CTGGGAGCTGCTGACTGCACA
cwp1F	ATGATGCTCGCTCTCCT
cwp1R	TCAAGGCGGGGTGAGGC
ranF	ATGTCTGACCCAATCAG
ranR	TCAATCATCGTCGGGAA
glycylrealF	TACTACAAGCATCAGGGC
glycylrealR	TAAGCTTATCTGCTCTGA
cwp1realF	AACGCTCTCACAGGCTCCAT
cwp1realR	AGGTGGAGCTCCTTGAGAAATTG
ranrealF	TCGTCCTCGTCGGAAACAA
ranrealR	AACTGTCTGGGTGCGGATCT
